# Responsible workplace data governance and organizational performance: evidence from Chinese listed firms

**DOI:** 10.3389/fpsyg.2026.1891689

**Published:** 2026-08-12

**Authors:** Lingtao Chen

**Affiliations:** School of Economics and Management, University of Electronic Science and Technology of China, Chengdu, China

**Keywords:** human-technology interaction, organizational performance, organizational trust, procedural fairness, responsible analytics, responsible workplace data governance, workplace transparency

## Abstract

**Background:**

Listed firms increasingly embed analytics, automated processing, algorithmic recommendations, and data flows into work, so data governance shapes how employees and managers experience authority, accountability, privacy, and fairness. This creates an urgent tension: data infrastructures can improve coordination and monitoring while obscuring how data are collected, interpreted, challenged, and audited, raising risks for trust, perceived fairness, voice, and cross-functional cooperation.

**Objective:**

Building on this tension and prior research that emphasizes responsible AI principles more than organizational evidence, this study examines whether employee-informed and disclosure-visible responsible workplace data governance is associated with organizational performance and whether governance friction, organizational learning, and technical complexity shape this association.

**Methods:**

Using 519 employee questionnaires from listed firms, five dimensions, and 19 indicators, this study builds a governance index and applies annual-report scoring to Chinese A-share non-financial firms from 2011 to 2023.

**Results:**

Two-way fixed effects estimates show that higher visible responsible workplace data governance is positively associated with ROA; a one-standard-deviation increase in the index corresponds to a 0.49 percentage point higher ROA, equal to 13.2% of the sample mean. An exploratory mediation check attributes 10.8% of this association to lower administrative compliance cost; organizational learning strengthens the association, technical complexity attenuates it, and evidence for Tobin's Q is positive but less stable across timing structures.

**Conclusion:**

For organizational psychology, the findings show that governance structures for data-intensive work can be measured at scale with employee-informed weights and behave as procedural-justice-relevant work-system conditions: they are associated with coordination-sensitive performance, strengthened by learning capability, and weakened by technical complexity, while the disclosure-based measure and observational design limit causal and individual-level psychological claims.

## 1 Introduction

Data-intensive and AI-enabled systems increasingly organize everyday work through analytics, automated processing, algorithmic recommendations, cross-functional data sharing, employee data management, customer records, supply-chain data, and audit trails. In Chinese listed firms, these systems are embedded in reporting, production, logistics, platform operations, human resources, and customer-facing processes, which means that data governance is experienced as a feature of work design rather than as a remote technical policy. Organizational psychology is therefore relevant because employees and managers need to understand how data are collected, how algorithmic outputs are reviewed, who can challenge them, and how responsibility is traced when data-driven decisions produce contested outcomes. Research on algorithmic management, big data ethics, and human-centered AI work design shows that data-intensive work changes the conditions under which people coordinate, evaluate fairness, and sustain trust in technology-mediated work systems (Jarrahi et al., 2021; Gal et al., 2020; Berretta et al., 2023).

Opaque workplace data practices create an organizational psychology problem before they create a technical one: when the rules of data collection, analysis, access, appeal, and audit remain unclear, employees lack the information that procedural fairness judgments and trust in work systems require, and coordination suffers as a result. Algorithmic control can reduce voice when workers perceive that data-driven rules are imposed without explainability or procedural fairness (Liang et al., 2026). Employee privacy research likewise shows that workplace datafication requires due diligence because the same data infrastructures that support monitoring and optimization can weaken human agency and rights protection (Ebert et al., 2021). People analytics research adds that ethical data use depends on whether organizations treat employees as participants in a governed work system rather than as passive data objects (Gal et al., 2020). These concerns make transparency, accountability, perceived fairness, privacy protection, and voice central to responsible analytics at work.

These workplace concerns also matter for organizational performance because trust, fairness, communication, and monitoring shape how employees use information and coordinate around tasks. Electronic performance monitoring research shows that employee reactions to data-based oversight depend on social exchange and justice perceptions (McNall and Roch, 2009). Work-performance research further indicates that employee perceptions of management systems are linked to workplace performance because monitoring practices can function as resources or demands depending on their design (de Menezes and Escrig, 2019; de Menezes and Escrig-Tena, 2023; Zhao et al., 2025). Human-AI team research similarly links trust and communication quality to team performance in AI-mediated collaboration (Chen and Zhang, 2025). These studies do not imply that a firm-level disclosure score directly measures employee trust or fairness, but they explain why visible governance routines may be relevant to downstream organizational performance. This chain from governance visibility through fairness-relevant and trust-relevant work conditions to organizational outcomes is the organizational psychology question this study operationalizes.

Responsible AI and workplace data ethics research provides the principle base for this governance problem. People analytics and workplace privacy studies highlight accountability, transparency, consent, and responsible use as core concerns in large-scale data processing (Gal et al., 2020; Ebert et al., 2021). Assessment-list research identifies technical robustness, transparency, privacy, fairness, human oversight, and accountability as evaluable requirements for trustworthy AI systems (Berretta et al., 2023). Corporate governance and AI ethics auditing research adds that principles require organizational procedures, audit trails, external assurance, and accountable implementation (Munz et al., 2023; Hiremath et al., 2025; Awan et al., 2026). The unresolved problem is measurement. Principle lists clarify what responsible governance should contain, but they do not automatically produce firm-year evidence that can be compared across organizations.

Organizational psychology explains how these governance practices become workplace-relevant. Transparent and accountable data routines can reduce uncertainty about how employee, customer, and operational data are used. Fairness and human oversight can support procedural justice because employees and managers have clearer grounds for contesting automated or data-driven decisions. Privacy and rights protection can reduce perceived vulnerability when data-intensive work expands. Learning climate and AI self-efficacy can also shape whether employees convert new data governance routines into usable practices (Wang et al., 2026; Shamim et al., 2023; Liang et al., 2026). These mechanisms position responsible workplace data governance as an organizational practice set that may support coordination and performance through trust-relevant and fairness-relevant work processes.

Large-sample evidence remains limited because the empirical object is difficult to observe. Direct surveys of trust, fairness, voice, and wellbeing provide valuable individual-level evidence, but they rarely scale across many firm-years. Annual reports are observable at scale, but they can capture symbolic disclosure as well as substantive practice. Machinewashing research warns that organizations may report favorable AI governance signals without matching routines (Calacci and Stein, 2023). Organizational implementation research nevertheless shows that responsible AI governance requires transparent control structures, accountable routines, and explicit implementation boundaries (Awan et al., 2026). A useful measure for organizational psychology must therefore combine employee-informed construct weighting with a disclosure-based firm-year proxy and must keep the measurement boundary explicit.

The Chinese listed-firm setting makes this measurement problem visible. Listed firms disclose annual reports with enough regularity to support panel analysis, while their digital transformation varies across industries, ownership structures, and technical capabilities. The same setting also requires caution because public disclosure may reflect regulatory pressure and reputation management. This study therefore asks whether responsible workplace data governance is associated with organizational performance among Chinese listed firms and whether learning capability, technical complexity, and governance friction condition that association. The scientific question does not treat visible disclosure as direct evidence that employees feel trust or fairness. It asks whether employee-weighted, annual-report visible governance practices are associated with downstream organizational performance in ways that are consistent with organizational psychology mechanisms and with clear evidence limits.

The study addresses this question through a two-stage measurement and panel design. First, 519 valid questionnaires from employees of listed companies are used to weight five dimensions and 19 indicators covering organizational governance, technical trustworthiness, rights protection, social impact, and operational mechanisms. Second, annual-report evidence is scored to construct a firm-year responsible workplace data governance index. The resulting index is matched with accounting, governance, and text-based variables for Chinese A-share non-financial listed firms from 2011 to 2023. The main outcome is return on assets, interpreted as downstream organizational performance. Tobin's Q is retained as secondary evidence of external recognition. Fixed effects, timing tests, dynamic panel estimates, and strengthened fixed effects improve the credibility of the association estimates without turning the design into a causal experiment.

The results support a ranked evidence structure. The strongest evidence is a positive association between responsible workplace data governance and ROA. A one standard deviation increase in the index corresponds to a 0.49 percentage point higher ROA, or 13.2 percent of the sample mean. Tobin's Q evidence is positive but weaker. The mechanism estimates indicate that higher governance scores are associated with lower compliance cost and that compliance cost is negatively associated with ROA, consistent with a partial governance-friction pathway. The moderation estimates show that organizational learning strengthens the governance-valuation relationship, while technical complexity weakens it. Ownership heterogeneity indicates stronger ROA associations among non-state-owned enterprises than among state-owned enterprises.

The study contributes an employee-informed firm-year measure and organizational evidence on responsible analytics governance at work. For organizational psychology specifically, it extends procedural justice and workplace monitoring research from individual perceptions toward the measurable governance structures that make those perceptions possible, and it does so at a scale that individual-level designs cannot reach. It links responsible workplace data governance to transparency, accountability, perceived fairness, trust-relevant coordination, learning capability, and organizational performance while keeping the survey and disclosure evidence separate. It also identifies boundary conditions that matter for implementation: learning capability helps firms turn governance principles into routines, while technical complexity increases auditability and verification burdens. The empirical contribution is therefore not a claim that annual reports reveal the full quality of workplace experience. It is a scalable measurement strategy for studying when visible governance routines, weighted by workplace-informed judgments, are associated with downstream organizational outcomes. This strategy gives organizational psychology a bridge between micro-level theories of trust, fairness, voice, and learning and a large panel setting in which organizational practices can be compared across firms and time.

## 2 Literature review

### 2.1 Responsible workplace data governance in data-intensive work

Responsible workplace data governance refers to visible organizational practices that make data-intensive work more transparent, accountable, privacy-respecting, fair, auditable, and open to human oversight. The construct draws from responsible AI, workplace datafication, and organizational psychology. Workplace datafication research adds that employee privacy and agency require human-rights-based due diligence because data collection in work settings involves power asymmetry between employer and employee (Ebert et al., 2021). Algorithmic management research shows that data systems reorganize managerial control, task allocation, monitoring, and evaluation in ways that change everyday work experience (Jarrahi et al., 2021; Huo et al., 2026; Won et al., 2023; Lang et al., 2023; Migliora, 2026).

The workplace setting makes governance more than a compliance concern. Employees interact with data systems through work instructions, monitoring dashboards, automated recommendations, customer analytics, production records, and performance measurement systems. When these systems are transparent and accountable, employees and managers can form shared expectations about evidence, review, responsibility, and appeal. When governance is vague, employees may treat data-driven outputs as arbitrary or strategically biased. Responsible governance therefore concerns the organizational routines that make data practices explainable and contestable inside work systems. These routines define organizational conditions under which data-intensive work remains trusted, fair, and coordinated.

The construct also clarifies why disclosure evidence can be useful despite its limits. Annual reports cannot reveal every lived employee experience, but they can show whether firms make governance responsibilities, privacy safeguards, rights-protection procedures, risk controls, and audit routines visible to employees, managers, and external audiences. Visibility matters in data-intensive work because employees and managers often coordinate through formal systems that require documentation and traceability. A disclosure-based measure therefore captures the publicly observable layer of workplace data governance. The measure is incomplete, but it remains informative when paired with employee-informed weighting and when interpretation stays within the boundary of visible practice.

### 2.2 Transparency, trust, fairness, and voice

Transparency and accountability matter because they shape whether employees perceive data-driven work systems as procedurally fair and open to voice. Perceived algorithmic control can discourage worker voice when employees believe that decisions are automated, opaque, or difficult to challenge (Liang et al., 2026). Electronic monitoring research similarly suggests that workers evaluate data-based oversight through social exchange and justice principles (McNall and Roch, 2009). Related studies on platform work, algorithmic control, transparency, and worker representation show that employee responses vary with perceived fairness, psychological empowerment, resources, and collective voice arrangements (Lin et al., 2025; Li et al., 2025; Tuomi et al., 2024; Collins and Atkinson, 2023; Gaudio, 2024). Trust in AI systems does not arise from technical performance alone. It depends on whether employees understand the system, can communicate about its outputs, and can rely on human and organizational safeguards when the system is wrong (Shamim et al., 2023; Chen and Zhang, 2025).

Fairness and voice also connect responsible governance to coordination. Data-intensive work often requires employees, departments, customers, and partners to share sensitive information. Opaque rules can increase verification demands because actors must spend additional effort checking whether data are accurate, whether access is legitimate, and whether decisions are reviewable. Transparent rules, clear accountability, human oversight, and appeal channels can reduce these frictions. This mechanism does not require the survey in this study to measure trust or fairness directly. The survey provides employee importance ratings for governance domains, while annual-report scoring captures visible practices. The organizational psychology link rests on prior theory and evidence showing why those visible practices are relevant to trust-relevant and fairness-relevant work systems.

Voice is especially important because data-intensive work often changes who can question a decision. When an allocation, evaluation, or recommendation comes from an analytic system, employees may treat the output as less contestable than a decision made by a supervisor. Governance routines can change this condition by defining review rights, escalation channels, and accountability for errors. The relevant psychological mechanism is procedural: employees are more likely to accept and use data systems when the organization makes the rules of explanation, correction, and responsibility visible. A firm-year disclosure score cannot observe acceptance directly, but it can record whether the organization reports the routines that make acceptance plausible.

### 2.3 Privacy, rights protection, and worker agency

Workplace data governance also requires privacy, consent, rights protection, and worker agency because data-intensive work often collects information about employees, customers, suppliers, and operations. Privacy due diligence research argues that employers must assess workplace big data practices through human rights and proportionality principles (Ebert et al., 2021). Collective data governance research adds that workers need access and understanding, not only abstract consent, if they are to participate meaningfully in data governance (Calacci and Stein, 2023). Research on people analytics, algorithmic surveillance, platform control, and worker rights shows that data-driven management can cross ethical boundaries when employees have little control over data reuse, profiling, automated evaluation, and legal recourse (Pawirosumarto and Kurniawan, 2026; Gal et al., 2020; Gaudio, 2024; Pesole, 2025; Liu et al., 2026).

These studies support the rights-protection and human-oversight dimensions in the index. Human oversight rights, user empowerment, fundamental rights protection, and protected reporting channels are not peripheral to workplace data governance. They define whether employees and other data subjects can question data use and whether organizations can trace responsibility. These practices can influence organizational coordination because unclear rights and weak appeals generate suspicion, contestation, and monitoring resistance.

### 2.4 Implementation, auditability, and visible governance measurement

Responsible governance requires implementation routines that can be assessed at scale. AI ethics research repeatedly finds a gap between stated principles and embedded organizational practice (Awan et al., 2026). AI governance studies likewise emphasize leadership control, autonomy, accountability, transparency, and strategic implementation as organizational conditions for responsible use (Hiremath et al., 2025; Madanchian and Taherdoost, 2025). AI ethics auditing research responds to this gap by emphasizing documentation, audit trails, independent review, and procedural assurance (Munz et al., 2023). Model-risk audit research similarly highlights reliability, explainability, privacy, and bias review as requirements for trustworthy deployment (Munz et al., 2023). These studies support a measurement strategy that looks for disclosed governance structures, technical safeguards, accountability mechanisms, risk management, and audit practices.

Visible disclosure remains imperfect. Strategic disclosure can produce favorable signals that are not matched by internal routines, a risk highlighted by machinewashing research (Calacci and Stein, 2023). Disclosure-based measurement can still contribute when it is treated as a bounded proxy for visible governance practice. Employee questionnaires inform the relative importance of dimensions and indicators. Annual-report scoring then provides a scalable firm-year measure of visible responsible workplace data governance. This two-part strategy does not fully observe lived employee experience, but it creates a transparent bridge between workplace-relevant governance constructs and large-sample organizational evidence.

### 2.5 Learning, technical complexity, and workplace effectiveness

Learning capability is central because responsible governance must be converted from abstract standards into routines, training, documentation, process redesign, and cross-functional coordination. Research on AI professionalism and responsible implementation suggests that organizations need leadership, foresight, and adaptive capacity to translate AI governance into practice (Awan et al., 2026; Berretta et al., 2023). Studies of algorithmic control, worker motivation, human-AI collaboration, and people analytics further show that technology-mediated work outcomes depend on whether employees receive resources, support, and learning conditions that help them adapt to data-driven systems (Gagne et al., 2022; Liu and Li, 2025; Jones et al., 2025; Wang et al., 2024). In workplace terms, learning capability should help employees and managers understand new data rules, update routines, and coordinate across technology, legal, human resources, and operating functions.

Technical complexity creates the opposite pressure. Complex data architectures, proprietary algorithms, and multi-layer systems make explainability, fairness auditing, and privacy protection harder to implement and verify. Research on human-AI collaboration task complexity shows that complexity changes employee engagement and requires supportive leadership and self-efficacy (Wang et al., 2026). Studies of algorithmic control also show mixed employee and performance responses when technical systems intensify monitoring, job demands, or psychological strain (Liang et al., 2024; Zhao et al., 2026; Mbare et al., 2024; Gao et al., 2025; Zhang et al., 2025). Requirements-engineering and audit research similarly suggests that complex AI systems increase the burden of documenting, reviewing, and validating responsible use (Munz et al., 2023; Hiremath et al., 2025). Organizational performance is therefore expected to depend not only on whether firms disclose governance practices but also on whether learning capability and technical complexity shape the conversion of those practices into workplace effectiveness.

Workplace effectiveness is treated here as a downstream organizational outcome rather than as an immediate psychological state. Prior work on monitoring and performance measurement shows that data-based systems can operate as resources when employees understand the system and as demands when monitoring increases pressure without control (McNall and Roch, 2009; de Menezes and Escrig-Tena, 2023). Responsible governance can help shift data systems toward the resource side by clarifying rules, responsibilities, and review channels. Technical complexity can push in the opposite direction because it makes those rules harder to operationalize. This tension motivates the empirical focus on learning and complexity as boundary conditions.

The performance link therefore runs through workplace effectiveness rather than through a narrow accounting logic. Data governance can make work more effective when it reduces uncertainty about data use, lowers coordination frictions, and supports shared expectations across functions. It can also fail to do so when disclosures remain symbolic, when employees cannot use the procedures, or when complex systems make accountability difficult. The empirical design cannot observe every step of this chain, but the literature review identifies why the chain is plausible and why the results must be interpreted within firm-level boundaries.

This literature position defines the level of inference. The analysis does not infer that every disclosed policy changes employee behavior. It asks whether a firm-year pattern of visible governance is associated with organizational outcomes in a direction consistent with workplace theories. The distinction matters because responsible data governance is both a formal organizational system and a lived work condition. Formal systems appear in disclosure, committees, policies, audits, and risk controls. Lived conditions appear in whether employees understand, trust, and use those systems. The study observes the formal layer and uses organizational psychology to explain why that layer should matter for workplace effectiveness.

## 3 Theory and hypotheses

### 3.1 Responsible workplace data governance and organizational performance

Three organizational psychology traditions explain why visible data governance should matter for workplace functioning. Procedural justice research shows that people evaluate decision systems by the fairness of their procedures, including consistency, correctability, and voice, and that these evaluations shape cooperation and performance (Colquitt et al., 2001). Uncertainty management research adds that fairness information becomes most consequential when employees face uncertainty about how a system will treat them, which is the default condition of algorithmic and data-driven decision processes (Lind and van den Bos, 2002). Monitoring research shows that data-based oversight functions as a resource when its rules are transparent and contestable and as a demand when they are not (McNall and Roch, 2009; de Menezes and Escrig-Tena, 2023). Responsible workplace data governance names the organizational practices that supply exactly these conditions: consistent documented procedures, correctable automated decisions, protected voice channels, and traceable accountability. The hypotheses that follow apply this reasoning at the level where the practices are institutionalized, the firm-year.

Responsible workplace data governance can be associated with organizational performance because it structures how employees and managers coordinate in data-intensive work. Transparent data practices clarify which data are collected, how they are processed, and how outputs are reviewed. Accountability practices identify responsibility for decisions and errors. Privacy protection and human oversight reduce uncertainty about inappropriate data use. These practices can lower verification demands, reduce disputes around data-driven decisions, and support trust-relevant coordination. At the firm level, better coordination can appear as higher operational profitability because resources are spent on productive work rather than on repeated checking, dispute management, or remediation. The association should be strongest for accounting performance because ROA reflects operating outcomes more directly than external market recognition.

This argument does not require a claim that the disclosure score directly measures trust or fairness. Instead, prior organizational psychology research explains why the indexed governance domains are relevant to workplace systems. The empirical test asks whether visible governance practices, weighted by employee importance ratings, are associated with downstream organizational performance after firm and year differences are controlled.

The expected association is also bounded by the type of outcome. ROA is an accounting-based organizational performance indicator that can reflect operating efficiency, coordination quality, and administrative burden. It is not a direct measure of employee performance or wellbeing. The study uses ROA because the available firm-year panel supports that outcome and because the theoretical argument concerns downstream organizational consequences of governance routines. This choice keeps the empirical test at the organizational level and avoids implying an unobserved individual-level pathway.

H1. Responsible workplace data governance is positively associated with organizational performance.

The expected association is also bounded by the visibility of the measure. Annual-report evidence captures what firms make observable about data governance, including responsibilities, safeguards, and audit routines. Visible governance can support performance because disclosure often reflects formalization, documentation, and cross-functional coordination. The same visibility can also reflect symbolic reporting. The hypothesis therefore concerns a positive association between visible governance and organizational performance after firm and year factors are absorbed. It does not require the stronger claim that every disclosed routine is implemented with equal quality inside the organization.

### 3.2 Governance friction and verification burden

Responsible governance may relate to performance partly through lower governance friction or verification burden. Data-intensive work creates friction when employees, managers, regulators, or partners must spend additional resources checking whether data processes are accurate, compliant, explainable, and fair. Clear data policies, audit trails, risk controls, human review, and accountability routines can reduce this burden by standardizing how decisions are documented and challenged. The available mediator is Compliance_Cost, measured as administrative expenses divided by operating revenue. This variable is not a direct psychological mediator. It is a partial administrative proxy for the costs that firms incur when governance, documentation, coordination, and compliance processes are inefficient.

Using the administrative expense ratio as a friction proxy follows an established precedent. Agency-cost research measures internal coordination and monitoring inefficiency with operating and administrative expense ratios, treating higher ratios as evidence of resources absorbed by oversight, documentation, and control problems (Ang et al., 2000; Singh and Davidson, 2003). The present argument uses the same logic in a narrower domain: when data governance clarifies responsibilities, documentation, and review channels, firms should spend less on the repeated checking and dispute management that appear in administrative expense.

The estimates support this logic. Higher governance scores are associated with lower compliance cost, and higher compliance cost is associated with lower ROA. This path captures only one part of the broader mechanism because trust, perceived fairness, voice, and coordination are not directly measured in the firm-year panel. It nevertheless tests whether visible responsible governance is linked to a lower administrative burden in a way that is consistent with the proposed workplace governance mechanism.

The mechanism claim is therefore deliberately narrow. A lower compliance-cost ratio can reflect fewer administrative frictions, better documentation, more standardized procedures, and lower verification burden. It can also reflect other unobserved managerial practices, and the ratio absorbs expenses unrelated to governance friction, including restructuring charges, managerial expansion, strategic project overhead, and administrative components of marketing and personnel programs, so the mediation estimate is informative only about the friction-related share of the association. The mediation test is useful because it checks whether the sign pattern is compatible with the governance-friction logic, not because it identifies the complete process. The estimated coefficient signs support the direction the theoretical argument requires.

H2. Governance friction or verification burden partially mediates the positive association between responsible workplace data governance and organizational performance.

The compliance-cost proxy has a narrow role in this argument. Administrative expense cannot capture all forms of verification burden, and it does not measure employee cognitive load. It can, however, reflect part of the organizational effort required to coordinate, document, review, and correct data-related processes. If responsible governance makes responsibilities and controls clearer, firms may spend fewer resources on repeated checking, remediation, and coordination failures. The mediation test is therefore interpreted as a partial pathway. A supported indirect path would indicate that lower administrative friction is consistent with the governance-performance association, while an unsupported path would require the mechanism to remain theoretical or move to the limitations. Because no direct measure of verification effort is available in the panel, the mediation analysis is best read as an exploratory consistency check on the friction pathway rather than as a confirmatory test of a psychological mechanism.

### 3.3 Organizational learning as a strengthening condition

Organizational learning capability should strengthen the association between responsible workplace data governance and performance because governance principles require interpretation, training, experimentation, and routine updating. Firms with stronger learning capability can scan external standards, absorb new knowledge, translate requirements into internal procedures, and coordinate changes across departments. Employees in such firms are also more likely to receive guidance that makes data governance usable in everyday work. These learning processes help transform visible policies into routines that support transparent data handling, accountable review, privacy protection, and cross-functional coordination.

The moderator is Org_Learning, a text-based proxy for training, research and development, knowledge management, and innovation language in annual reports. It is best interpreted as a firm-level organizational learning capability proxy, not as a direct measure of employee learning climate. The expected interaction is positive because governance practices should yield larger organizational benefits when firms have stronger capability to implement and update them.

This boundary condition also protects the baseline hypothesis from a universal-performance claim. Responsible governance practices should be more useful when firms can absorb new standards and make them actionable. Without learning capability, firms may disclose policies but fail to convert them into usable work routines. The theory therefore expects the governance relationship to vary with implementation capacity rather than to appear uniformly across all firms.

H3. Organizational learning capability strengthens the positive association between responsible workplace data governance and organizational performance.

Learning capability matters because responsible data governance often requires employees and managers to update routines at the same time. A new privacy rule, model-review procedure, or appeal channel changes work only when people know the rule, understand when it applies, and can coordinate with others who share responsibility for the data process. Learning-oriented firms are more likely to routinize training, document lessons from incidents, and revise procedures when technology or regulation changes. The same governance disclosure should therefore carry more organizational value when the firm has stronger learning signals, because employees and managers are better able to convert formal rules into usable work practices.

### 3.4 Technical complexity as a weakening condition

Technical complexity should weaken the association between responsible workplace data governance and performance because complex systems are harder to explain, audit, and govern. Firms with high technical complexity may use multi-layer architectures, proprietary algorithms, and specialized data infrastructures. These features can improve operations, but they also raise the costs of tracing decisions, documenting data lineage, testing fairness, protecting privacy, and assigning accountability. Employees and managers may face greater difficulty understanding how outputs are produced and how errors can be challenged. External observers may also discount governance disclosures when the underlying systems are hard to verify.

The moderator is Tech_Complexity, a composite of firm research and development intensity and the digitization level of the firm's city, described in the Methods section. The estimated interaction is negative, which supports the attenuation logic. The hypothesis remains bounded by the proxy. The composite captures implementation complexity of the firm's technical environment rather than direct psychological experience of complexity at work. The expected pattern is that the same level of visible governance is less strongly associated with performance in technically complex settings because auditability and implementation burdens are higher.

This hypothesis also explains why technical capability and governance returns may diverge. Complex systems can create valuable operational capabilities, but they can also make responsibility tracing, privacy review, model explanation, and fairness testing more demanding. The empirical prediction concerns this second channel. If complexity raises the cost of making governance credible and usable, then visible governance practices should be less strongly associated with performance under high technical complexity.

**Figure 1:**
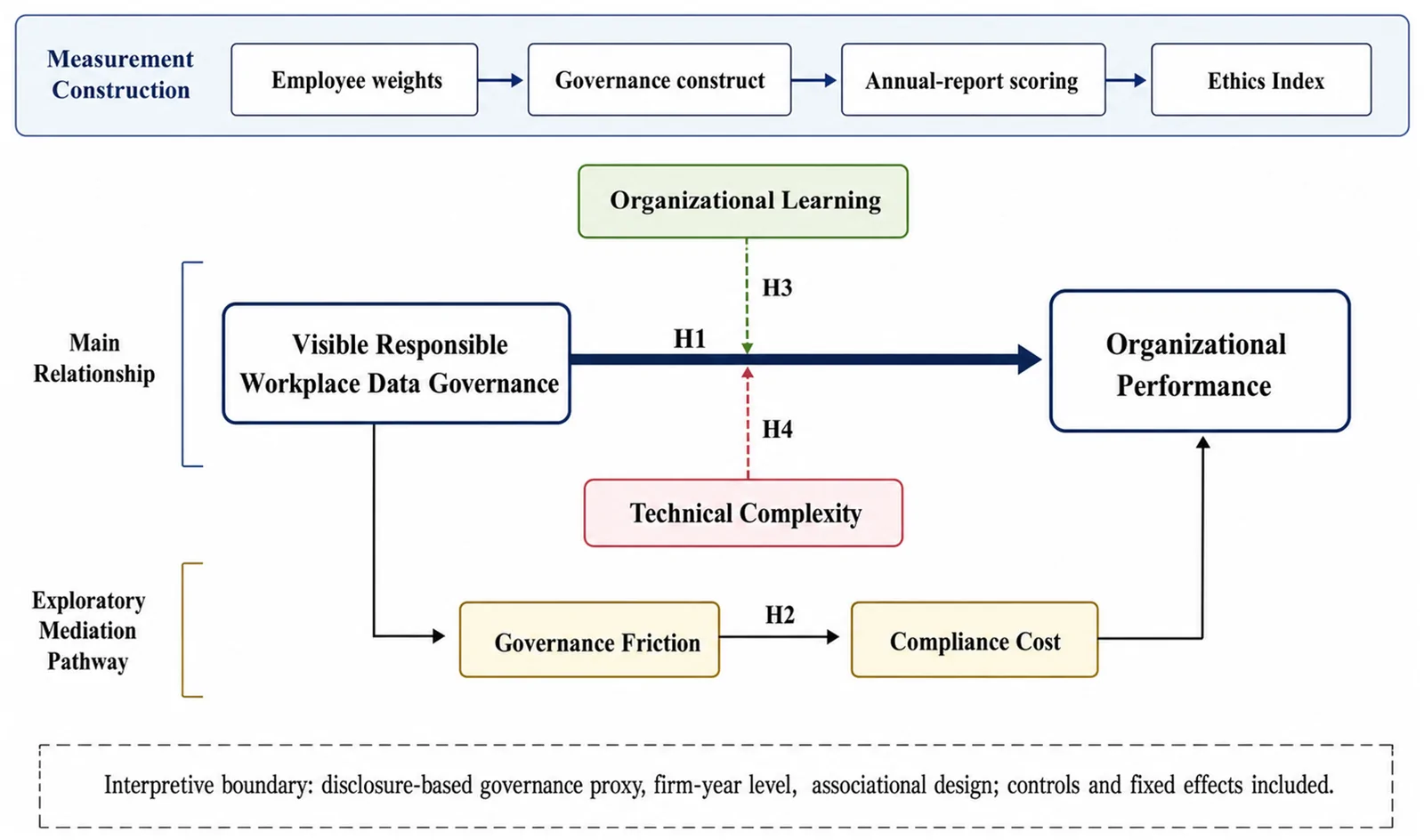
Responsible workplace data governance is linked to downstream organizational performance through lower governance friction, with organizational learning and technical complexity shaping the strength of the relationship.

The four hypotheses are therefore ordered from the most general association to more conditional claims. H1 establishes whether visible responsible workplace data governance is associated with organizational performance. H2 tests one observable friction pathway. H3 and H4 test whether implementation capacity and technical burden alter the relationship. Figure 1 summarizes this conceptual structure by linking visible governance to organizational performance through governance friction, with organizational learning and technical complexity specifying the main boundary conditions. This ordering allows the Results section to report the strongest evidence first and to qualify the mechanism and boundary evidence according to the estimation results.

H4. Technical complexity weakens the positive association between responsible workplace data governance and organizational performance.

## 4 Materials and methods

### 4.1 Research setting and sample

The empirical setting is Chinese A-share non-financial listed firms from 2011 to 2023. This setting provides a large organizational population in which data-intensive technologies, disclosure obligations, and workplace governance practices developed during a period of rapid digital transformation. Financial and insurance firms are excluded because their regulatory treatment, capital structure, and performance measures differ from those of industrial and service firms. Firms with abnormal listing status and observations with missing core variables are removed. Continuous variables are winsorized at the 1st and 99th percentiles to reduce the influence of extreme values. The analysis uses annual-report disclosure, employee questionnaire evidence, and firm-year financial and governance data. Table 1 traces the sample from the initial firm-year pool to the estimation sample and reports the observations removed at each exclusion step; models with additional data requirements lose further observations, and each results table reports its own sample size.

**Table 1:** Sample construction.

Step	Removed	Remaining
Initial A-share firm-year pool, 2011–2023		52,847
Financial and insurance industry firms	1,892	50,955
ST, ^*^ST, and PT listing status	2,134	48,821
Governance index not constructable	12,340	36,481
Missing outcome or control variables	8,934	27,547
Final estimation sample		27,547

The descriptive statistics in Table 2 use the 36,481 firm-years with a constructable governance index. Models that require lags, leads, or moderators lose additional observations, and each results table reports its own sample size.

The sample is interpreted as an organizational setting rather than as a capital-market sample. Chinese listed firms offer observable annual reports and firm-year outcomes, which makes it possible to compare visible data governance practices across organizations. The results should not be generalized to unlisted firms, other jurisdictions, or individual employee outcomes without additional evidence. The fixed effects models compare within-firm changes over time while absorbing firm-invariant differences and common year shocks. This design supports conditional association claims and robustness analysis but does not establish definitive causality.

The exclusion rules also protect the organizational interpretation of the outcomes. Financial and insurance firms operate under sector-specific prudential rules, reporting requirements, and balance-sheet structures that would make ROA and governance disclosure less comparable with other firms. Excluding abnormal listing-status firms reduces the chance that distress, delisting pressure, or restructuring dominates the governance-performance relationship. The resulting sample is therefore designed for comparability across ordinary listed organizations.

### 4.2 Employee questionnaire and indicator weighting

The survey instrument was the Corporate Big Data Ethics Indicator Importance Assessment Questionnaire. It was administered to employees and professionals connected to listed-company work contexts and produced 519 valid questionnaires after excluding invalid or logically inconsistent responses. The questionnaire collected respondent characteristics, direct weight allocation across five first-level dimensions, five-point Likert importance ratings for 19 second-level indicators, keyword evaluations for annual-report text analysis, indicator rankings, evaluation-method rankings, and suggested pass or excellence thresholds for a 100-point index.

The questionnaire was administered through the Wenjuanxing platform's professional sample service between 20 and 27 June 2025 in a single cross-sectional wave, targeting currently employed adults in China. A dedicated consent page stated the research purpose, anonymity, exclusive academic use, and voluntary participation, and respondents proceeded only after confirming agreement. Of 847 collected responses, 328 were excluded through sequential screening rules covering response times below 180 s or above 1,800 s, a failed attention-check item, dimension-weight allocations that did not sum to 100 percent within a five-point tolerance band, and straight-lined answer blocks, which produced the 519 valid questionnaires.

The respondent profile supports employee-informed weighting. The extracted questionnaire report shows 46.24 percent male and 53.76 percent female respondents. Respondents aged 31–40 account for 59.15 percent, and bachelor's degree holders account for 80.54 percent. Non-managerial professional roles account for 71.87 percent, while 40.08 percent report 3–5 years of data-management or digitalization experience and 26.97 percent report 6–10 years. Industry coverage includes internet platforms and services, intelligent manufacturing, e-commerce and retail, education technology, healthcare services, telecommunications, and energy and basic materials.

The questionnaire also records how respondents evaluate the measurement approach. Government compliance inspection, third-party field audit, annual-report text analysis, respondent questionnaire evidence, media sentiment, and combined methods are all ranked as possible evaluation sources. This evidence is useful because it shows that annual-report scoring is one recognized measurement route, but it also shows why the study should not treat disclosure as a complete implementation audit. The final index therefore uses annual reports for scalable scoring and keeps the questionnaire as the source of employee-informed weighting rather than as a substitute for direct workplace observation.

The survey is used only for importance weighting and measurement calibration. It does not measure organizational trust, perceived fairness, wellbeing, employee voice, or psychological states. Respondents rated the importance of governance dimensions and indicators for evaluating responsible data governance, which this study treats as employee-informed responsible workplace data governance. The final dimension weights are technical trustworthiness, 25.69 percent; organizational governance, 21.58 percent; rights protection, 18.89 percent; operational mechanisms, 17.09 percent; and social impact, 16.76 percent. Mean Likert scores are 4.12, 4.04, 4.04, 4.15, and 3.95 for these dimensions, respectively.

The survey weights are cross-sectional, so their application to a multi-year panel requires justification. Respondents evaluated the relative importance of governance dimensions and indicators rather than the practices of any particular firm-year. The weights therefore encode a judgment structure about what responsible data governance consists of, and this structure enters the index as a set of scaling constants that are identical for every firm and every year. Within-firm estimates are consequently unaffected by the level of the weights; a threat arises only if the relative importance ordering itself shifted materially over the study period. Two features limit this threat. First, 67 percent of respondents report three to ten years of data-management experience, so the ratings reflect judgments formed across a substantial part of the sample window rather than a single point in time. Second, and more directly, the results section reports weight-perturbation tests showing that the main associations are insensitive to the specific weight vector, together with a recent-period subsample that excludes the earliest sample years.

### 4.3 Responsible workplace data governance index construction

The index contains five first-level dimensions and 19 second-level indicators. Organizational governance includes ethics awareness maturity, leadership commitment, cross-departmental collaboration, and policy completeness. Technical trustworthiness includes technical robustness, privacy and data governance, transparency, and algorithmic fairness. Rights protection includes human oversight rights, user empowerment, fundamental rights protection, and whistleblower system effectiveness. Social impact includes social welfare, environmental impact, employment impact, and malicious-use prevention. Operational mechanisms include accountability mechanisms, risk management, and ethics compliance auditing.

The five dimensions are retained because they capture distinct aspects of responsible workplace data governance. Organizational governance identifies whether responsibility and coordination structures exist. Technical trustworthiness identifies whether data systems are robust, privacy-preserving, transparent, and fair. Rights protection identifies whether affected persons have oversight, empowerment, rights safeguards, and reporting channels. Social impact records whether firms attend to broader work and community consequences. Operational mechanisms identify whether accountability, risk management, and audit routines make governance enforceable. Keeping all dimensions preserves the original measurement richness while making the construct more relevant to workplace data systems.

Annual reports were machine-coded against a predefined keyword dictionary; no discretionary manual scoring entered the firm-year index. The corpus consists of the full annual reports of Chinese A-share non-financial listed firms from 2011 to 2023, downloaded from the CNINFO disclosure platform and converted to plain text, with the Management Discussion and Analysis section extracted through section-header pattern matching. Section headers, tables of contents, audit-opinion paragraphs, financial-statement tables, and page artifacts were stripped before analysis. The text was segmented with the jieba Python package, version 0.42.1, Sun Junyi/jieba project, China, https://github.com/fxsjy/jieba and the Harbin Institute of Technology stop-word list, augmented with report-specific boilerplate terms, was applied.

The dictionary was built from the questionnaire rather than from researcher judgment alone. The survey's keyword-evaluation task asked all 519 respondents to rate how well each candidate keyword represented responsible data governance practices on a five-point scale, and keywords with a mean rating below 3 were excluded. The final dictionary contains 57 keywords mapped to the 19 second-level indicators; the full keyword-to-indicator mapping appears in Supplementary material. Each keyword was treated as an independent search term. The dictionary does not perform synonym expansion or negation filtering, so the index measures the volume of governance-related disclosure rather than its directional valence, a design that follows established practice in disclosure-based text measurement for Chinese listed firms (Wu et al., 2021) and whose implications are addressed in the limitations.

For each firm-year report, the indicator score equals the frequency of that indicator's dictionary keywords per 10,000 segmented words, which removes differences in report length. Indicator scores were averaged with equal weights within each dimension to produce the five dimension scores. Dimension scores were then aggregated to the firm-year index using hybrid weights that combine the survey's direct dimension-weight allocations with entropy-based information content in equal proportions, yielding the final dimension weights reported above. The composite was rescaled to a 0 to 100 range using the full-sample minimum and maximum. As a concrete illustration, a disclosure stating that the firm has established a data security and compliance committee led by a named executive and conducts quarterly audits of algorithmic fairness and privacy compliance contributes keyword hits to leadership commitment, ethics compliance auditing, algorithmic fairness, and privacy and data governance; worked examples from actual reports, including a generic-disclosure negative case that receives no credit, appear in Supplementary material.

Because dictionary-based machine coding can misread context, a stratified random validation subsample of 120 firm-year reports, allocated proportionally across years and one-digit industries, was coded independently by two trained coders. Both coders completed a 4-h training program using the written coding manual, which defines each of the 19 indicators, provides positive and negative anchor examples, and specifies boundary rules; twenty pilot reports outside the validation sample were used to calibrate the manual. Inter-rater reliability reached a mean Cohen's kappa of 0.726 across indicators, with dimension-level values between 0.691 and 0.758, above the conventional 0.61 threshold for substantial agreement. Remaining disagreements were resolved through structured consensus, and the eight indicator-level judgments (0.35 percent) that resisted consensus were settled by the author. The coding manual excerpt and validation details appear in Supplementary material.

The resulting score is a disclosure-based firm-year proxy for visible responsible workplace data governance rather than a direct observation of internal routines or employee experience. A high score indicates that annual reports contain more structured evidence of governance routines, technical safeguards, rights protection, social-impact consideration, and operational accountability mechanisms. Observed values concentrate in the lower part of the 0 to 100 scale because listed firms disclose limited and conservative information about data governance; this concentration affects interpretation of absolute values but still permits comparison of relative variation across firms and years.

### 4.4 Dependent variables, mediator, moderators, and controls

The primary dependent variable is return on assets, defined as net income divided by average total assets. This study interprets ROA as downstream organizational performance because it reflects operating outcomes that may be associated with coordination, verification burden, and implementation efficiency. Tobin's Q is retained as a secondary outcome and is interpreted as external market recognition. The analysis uses the natural logarithm of (Tobin's Q + 1) to reduce skewness. The secondary status of Tobin's Q is important because market valuation is more sensitive to investor attention, disclosure credibility, and information absorption.

Compliance_Cost is measured as administrative expenses divided by operating revenue. It is interpreted as a partial proxy for governance friction or verification burden when the estimated signs support that logic. Org_Learning is a text-based proxy for organizational learning capability constructed from the same annual-report corpus. A learning-oriented dictionary of 50 terms, compiled from the organizational learning measurement literature and adapted to Chinese corporate reporting, covers organizational learning and knowledge management, employee training and human capital development, innovation absorption, and continuous improvement; the full term list appears in Supplementary material. For each firm-year, the measure equals the frequency of dictionary terms per 10,000 segmented words in the Management Discussion and Analysis section, standardized to zero mean and unit variance over the 16,612 firm-year observations with usable text. Missing coverage reflects report availability and optical-recognition quality screening rather than firm characteristics of theoretical interest. Models that require the full panel use an imputed version in which missing values are set to zero, the mean of the standardized score; the moderation result does not depend on this imputation, because re-estimating the learning moderation on the text-covered subsample alone leaves the interaction positive and marginally significant (b = 0.0051, *t* = 1.65, *N* = 16,612). Convergent evidence supports the construct interpretation: the measure correlates at 0.50 with annual-report digitalization intensity and at 0.22 with patenting activity, consistent with learning-related disclosure rather than generic report length. Tech_Complexity combines firm research and development intensity, measured as research and development expenditure divided by operating revenue in percent, with the city-level digitization index of the firm's registered location: the two are averaged when both are available, and the city-level index substitutes when firm research and development intensity is missing. The composite captures the technical complexity of the firm's implementation environment rather than research and development spending alone, and it enters the moderation models as a standardized score, so its scale does not affect the estimates. Controls include firm size, leverage, board size, independent director ratio, ownership concentration, firm age, financing constraints measured by the Hadlock and Pierce size-age index, industry concentration measured by a revenue-based Herfindahl-Hirschman index, and digital transformation intensity measured by text-mined digital keyword frequency. These controls absorb observable differences in scale, capital structure, governance, ownership, lifecycle, financing conditions, competitive structure, and digitalization, and the digitalization control separates visible governance from generic digital adoption.

### 4.5 Model specification and estimation

The baseline model estimates the association between responsible workplace data governance and organizational performance using two-way fixed effects: The baseline specification is presented in Equation 1.


$$\begin{array}{c} Performance_{it}=\beta_{0}+\beta_{1}Governance_{it}+\underset{j}{\sum }\beta_{j}Controls_{it}+\mu_{i}+\lambda_{t}+\varepsilon_{it}. \end{array}$$ (1)

Here, *Performance*_*it*_ is ROA or lnTobinQ for firm *i* in year *t*, *Governance*_*it*_ is the responsible workplace data governance index, *Controls*_*it*_ is the control vector, μ_*i*_ is the firm fixed effect, and λ_*t*_ is the year fixed effect. Standard errors are clustered at the firm level. The coefficient β_1_ is interpreted as a within-firm association after stable firm characteristics and common year shocks are absorbed. The equation is written with firm fixed effects, and the ROA models are estimated in exactly this form. The valuation models replace the firm effects with industry effects while keeping year effects, because variation in lnTobinQ is dominated by market-wide and sector-level revaluation; the industry and year structure absorbs those components while preserving cross-firm comparison within industries. Every results table states the fixed-effects structure it uses.

Moderation tests add the moderator and interaction term. Mediation tests follow a stepwise structure and include a Sobel test and bootstrap confidence interval for the indirect path. Robustness checks include alternative profitability outcomes, lagged governance specifications, difference GMM, lead-placebo tests, distributed-lag tests, and strengthened fixed effects using province-by-year or industry-by-year structures. These procedures strengthen the association evidence and reduce several plausible threats, but they do not eliminate all time-varying omitted variables or reverse causality.

The empirical design also preserves the hierarchy of evidence. Baseline ROA models are the primary performance tests. Tobin's Q models are secondary because external recognition depends on whether governance disclosures are noticed and considered credible. Mediation and moderation models are interpretive tests that examine whether the data match the proposed friction and boundary-condition logic. Robustness tests evaluate sensitivity rather than create a new claim. This hierarchy keeps the Results section from treating all estimates as equally strong.

### 4.6 Measurement boundaries

The measurement design has five boundaries. First, the survey supports employee-informed weights and indicator importance, not individual-level trust, fairness, voice, wellbeing, or learning-climate outcomes. Second, annual-report scoring measures visible practices and disclosed routines, not full internal implementation. Third, compliance cost is an administrative proxy for governance friction or verification burden, not a direct measure of employee cognitive burden or psychological strain. Fourth, ROA and Tobin's Q are organizational outcomes. They can be interpreted as downstream indicators of workplace effectiveness and external recognition, but they cannot identify the full psychological process linking governance to employee attitudes. Fifth, the keyword dictionary counts governance-related disclosure without synonym expansion or negation filtering, so the index measures the volume of visible governance language rather than its directional valence. These boundaries are carried through the Results and Discussion sections.

## 5 Results

### 5.1 Measurement and descriptive evidence

The first empirical question is whether the responsible workplace data governance index has enough variation to support firm-year analysis. Table 2 reports descriptive statistics for the core variables. The sample contains more than 36,000 firm-year observations for the descriptive variables. The governance index has a mean of 13.893 and a standard deviation of 4.430, with values ranging from 2.754 to 25.140. The observed range indicates meaningful variation in annual-report visible governance practices, although the low absolute level confirms that disclosure remains sparse.

**Table 2:** Descriptive statistics for governance, performance, and boundary variables.

Variable	*N*	Mean	Std. Dev.	Min	Max
ROA	36,481	0.037	0.065	–0.274	0.205
lnTobinQ	35,889	1.038	0.332	0.613	2.275
Responsible governance index	36,481	13.893	4.430	2.754	25.140
Organizational learning	36,481	–0.005	0.627	–1.721	2.507
Technical complexity	36,481	137.813	68.233	0.080	326.222
Compliance cost	36,481	0.491	0.588	0.004	3.735
Size	36,481	22.272	1.438	19.785	27.321
Leverage	36,481	0.391	0.256	0.000	0.898
Financing constraints (SA index)	36,481	–3.808	0.280	–4.436	–2.754
Industry concentration (HHI)	36,481	0.197	0.179	0.000	1.000
Digital transformation intensity	36,481	1.490	1.420	0.000	5.165

The descriptive statistics show that ROA averages 3.7 percent with a standard deviation of 6.5 percent. lnTobinQ averages 1.038. Compliance cost averages 49.1 percent of operating revenue, while technical complexity has large dispersion because research and development intensity and city digitization levels differ sharply across industries and locations. Supplementary correlations show that the governance index is positively correlated with ROA (*r* = 0.159, *p* < 0.01) and negatively correlated with compliance cost (*r* = −0.166, *p* < 0.01). Its raw correlation with lnTobinQ is negative (*r* = −0.092, *p* < 0.01), a pattern explained by size confounding because larger firms tend to disclose more governance practices while receiving lower valuation multiples.

The descriptive evidence also supports the measurement boundary. The index is not close to its theoretical maximum, which is consistent with sparse disclosure rather than with full implementation of responsible data governance. The mean value should therefore not be interpreted as an absolute level of workplace trustworthiness. Its use lies in cross-firm and within-firm variation. Firms with higher scores disclose more visible practices related to governance, rights protection, technical safeguards, and auditability. The empirical models test whether this variation is associated with organizational outcomes after the main controls and fixed effects are included.

The measurement question also requires evidence about who provided the employee-informed weights. Table 3 reports the respondent profile from the questionnaire report. The profile shows that the survey primarily reflects working-age employees and professionals in data-intensive organizational settings. Non-managerial professional roles account for 71.87 percent of the 519 valid responses, while grassroots managers account for another 17.53 percent. Data-management experience is concentrated among respondents with 3 to 5 years and 6 to 10 years of experience. This profile supports the use of the questionnaire as workplace-informed importance evidence.

**Table 3:** Questionnaire respondent profile for employee-informed measurement.

Profile item	Largest or reported categories	Valid responses
Gender	Female 53.76%; male 46.24%	519
Age	31–40 years 59.15%; below 30 years 29.48%	519
Education	Bachelor's degree 80.54%; master's degree 13.87%	519
Role level	Non-managerial professional roles 71.87%; grassroots managers 17.53%	519
Data-management experience	3–5 years 40.08%; 6–10 years 26.97%; below 3 years 25.63%	519
Firm size	Below 500 employees 50.10%; 500–2,000 employees 35.84%	519
Industry	Internet platforms and services 24.66%; intelligent manufacturing 24.28%; e-commerce and retail 15.22%	519
Data-governance participation	Important participant 10.21%; primary responsible person 2.70%; occasional participant 12.52%	519

Percentages are taken from the questionnaire report. The profile supports interpretation of the weights as employee and professional importance ratings, not as direct workplace-experience outcomes.

The respondent profile also helps interpret the five-dimensional weighting structure. Respondents are close enough to listed-company data work to evaluate the importance of transparency, privacy, technical robustness, rights protection, and accountability, but the survey design asked them to rate governance indicators rather than their own attitudes. The index therefore combines workplace salience with annual-report observability. This distinction is important for the rest of the evidence sequence. The descriptive table shows firm-year variation in disclosed practices, while the profile table shows why the weights can be treated as employee-informed. Neither table by itself establishes that employees inside high-scoring firms experience higher trust or fairness.

The two measurement tables should be read together. Table 2 shows that the archival index varies enough to support panel estimation, while Table 3 shows that the weighting evidence is anchored in respondents with relevant work and data-management experience. The tables also prevent two possible overreadings. The low mean score does not mean that Chinese listed firms lack all responsible practices, because the measure records visible annual-report evidence.

Construct validity was examined directly. In the full estimation sample, the governance index correlates at 0.38 with third-party ESG ratings, 0.42 with text-mined digital transformation intensity, and 0.19 with patenting activity, which supports convergent validity while remaining well below levels that would indicate construct redundancy; Supplementary material reports the full matrix. Discriminant validity was tested with horse-race regressions. Table 4 shows that the governance coefficient remains positive and significant at the 1 percent level when ESG ratings, digital transformation intensity, and patent intensity enter the ROA model individually and jointly (b = 0.0008, t = 4.10 in the full specification). The index therefore carries information about visible responsible data governance beyond adjacent disclosure constructs, which addresses the concern that annual-report scoring mainly captures generic disclosure quality or symbolic reporting.

**Table 4:** Discriminant validity: horse-race regressions for ROA.

Variable	(1)	(2)	(3)	(4)
Responsible governance index	0.0011*** (6.13)	0.0008*** (3.75)	0.0013*** (6.48)	0.0008*** (4.10)
ESG rating (z)		0.0049*** (4.95)		0.0042*** (4.22)
Digital transformation (z)			-0.0046*** (-5.32)	-0.0042*** (-4.82)
Patent intensity (z)				0.0013** (2.08)
Controls	Yes	Yes	Yes	Yes
Fixed effects	Firm and year	Firm and year	Firm and year	Firm and year
Observations	27,547	27,547	27,547	27,547
Adjusted *R*^2^	0.123	0.128	0.126	0.131

t-statistics are reported in parentheses. Standard errors are clustered at the firm level. ESG rating is the third-party composite rating, digital transformation is the text-mined intensity measure, and patent intensity is logged patent applications, each standardized. Columns (3) and (4) omit the digital transformation control variable because the standardized competing construct replaces it. *** *p* < 0.01, ** *p* < 0.05.

### 5.2 Baseline association with organizational performance

The next question is whether visible responsible workplace data governance is associated with organizational performance after observable controls and fixed effects are included. Table 5 reports baseline estimates for ROA and lnTobinQ. The ROA model uses firm and year fixed effects, while the valuation model uses industry and year fixed effects.

**Table 5:** Responsible workplace data governance and organizational performance.

Variable	(1) ROA	(2) lnTobinQ
Responsible governance index	0.0011*** (6.13)	0.0025*** (3.17)
Size	0.0147*** (9.60)	–0.1228*** (–29.01)
Leverage	–0.0612*** (–16.45)	–0.1350*** (–8.89)
Board size	–0.0004 (–0.60)	0.0023 (1.05)
Independent directors	–0.0215 (–1.40)	0.1906*** (3.01)
Top1	0.0006*** (6.48)	0.0001 (0.18)
Age	–0.0161*** (–9.80)	0.0916*** (17.91)
Financing constraints (SA index)	–0.0226** (–2.57)	0.2218*** (12.86)
Industry concentration (HHI)	–0.0079* (–1.88)	0.0251 (1.08)
Digital transformation intensity	–0.0034*** (–4.59)	0.0051* (1.65)
Constant	–0.3061*** (-6.52)	4.3900*** (34.15)
Fixed effects	Firm and year	Industry and year
Observations	27,547	27,030
Adjusted *R*^2^	0.123	0.411

t-statistics are reported in parentheses. Standard errors are clustered at the firm level. *** *p* < 0.01, ** *p* < 0.05, * *p* < 0.10.

Column (1) shows that the governance index is positively associated with ROA. The coefficient is 0.0011 with a t-statistic of 6.13. A one-unit increase in the index corresponds to a 0.11 percentage point higher ROA. A one standard deviation increase in the index, equal to 4.43 units, corresponds to a 0.49 percentage point higher ROA. Relative to the mean ROA of 3.7 percent, this difference is 13.2 percent of the sample mean. This estimate is the primary organizational performance evidence because ROA reflects operating outcomes more directly than market valuation.

The magnitude sits in the small-to-moderate range typical of governance-related disclosure measures. A one standard deviation difference in the index corresponds to 0.49 percentage points of ROA, or 13.2 percent of the sample mean, and the same difference corresponds to 1.15 percentage points of ROE in the robustness models reported below. Meta-analytic evidence on ESG and corporate financial performance finds mostly positive but modest associations across thousands of estimates (Friede et al., 2015), and governance-quality research reports economically meaningful but similarly bounded performance differences between well-governed and poorly governed firms (Gompers et al., 2003). The estimate reported here is therefore plausible for a disclosure-based organizational practice measure: large enough to matter for operating performance and small enough to be consistent with an indirect coordination pathway rather than a mechanical accounting effect.

Column (2) reports the secondary valuation evidence. The governance coefficient is 0.0025 and significant at the 1 percent level. The positive sign suggests that visible governance may be recognized externally, but the timing tests reported below still require caution before treating valuation as primary evidence. Investors must observe, process, and trust disclosure-based governance information before incorporating it into valuation. This additional absorption process makes Tobin's Q a secondary and more conditional outcome rather than the core evidence for the organizational psychology argument.

The control variables support the interpretation that the governance coefficient is estimated after important organizational differences are absorbed. Firm size is positively associated with ROA but negatively associated with lnTobinQ, which helps explain why the raw governance-valuation correlation differs from the conditional coefficient. Leverage is negatively associated with both outcomes. Firm age is negatively associated with ROA and positively associated with valuation, while ownership concentration shows a small positive ROA coefficient. Financing constraints, industry concentration, and digitalization intensity absorb further structural differences across firms. These controls do not eliminate all confounding, but they reduce the chance that the governance estimate is simply a proxy for scale, capital structure, board composition, ownership, or lifecycle.

The baseline table therefore establishes the main evidence hierarchy used throughout the article. ROA provides the core operating-performance association, while Tobin's Q provides a weaker external-recognition check. The coefficient pattern is consistent with the idea that visible governance is closer to internal coordination and administrative efficiency than to immediate market valuation. Later robustness and timing tables test whether this hierarchy survives alternative specifications.

### 5.3 Governance friction and verification burden

The mechanism question is whether a lower governance-friction proxy accounts for part of the governance-performance association. Table 6 reports the mediation estimates. Higher governance is associated with lower compliance cost, and higher compliance cost is associated with lower ROA.

**Table 6:** Governance friction and compliance-cost mediation.

Variable/statistic	(1) Compliance cost	(2) ROA
Responsible governance index	–0.0038*** (–3.27)	0.0010*** (5.54)
Compliance cost		–0.0313*** (–13.88)
Controls	Yes	Yes
Fixed effects	Firm and year	Firm and year
Observations	27,547	27,547
Adjusted *R*^2^	0.1337	0.1030
Sobel test z-statistic	3.12***	
Bootstrap 95% CI, indirect path	[0.00008, 0.00019]	
Mediation proportion	10.8%	

t-statistics are reported in parentheses. Bootstrap uses 5,000 replications. *** *p* < 0.01.

Column (1) indicates that a one-unit higher governance score is associated with a 0.0038 lower compliance-cost ratio. A one standard deviation higher governance score corresponds to a 1.68 percentage point lower administrative expense ratio, or a 3.4 percent difference relative to the compliance-cost mean. Column (2) shows that compliance cost is negatively associated with ROA. The governance coefficient remains positive and significant after compliance cost is included, declining from 0.0011 in the baseline model to 0.0010. The Sobel statistic and bootstrap interval support a non-zero indirect path, and the estimated mediation share is 10.8 percent.

This evidence is consistent with a partial governance-friction pathway. It should not be read as a complete psychological mechanism. Compliance cost is an administrative measure, so it captures only part of the verification and coordination burden that data-intensive governance may reduce. The remaining association may involve other channels such as trust-relevant transparency, fewer disputes about data use, stronger coordination routines, or reputation. The data do not directly observe these channels at the individual level.

The result also clarifies how H2 should be stated. The original compliance-cost language can be retained only as governance friction or verification burden. The estimated signs support that interpretation because stronger governance is associated with lower cost and lower cost is associated with higher ROA.

### 5.4 Learning and technical-complexity boundary evidence

The boundary-condition question is whether the governance association is stronger when firms have more learning capability and weaker when technical complexity is high. Table 7 reports the moderation estimates. The estimates show a positive interaction for organizational learning and a negative interaction for technical complexity.

**Table 7:** Organizational learning and technical complexity as boundary conditions.

Variable	(1) lnTobinQ	(2) lnTobinQ
Responsible governance index	0.0064* (1.84)	0.0081** (2.30)
Organizational learning	0.0223*** (3.87)	
Governance × organizational learning	0.0049* (1.69)	
Technical complexity		0.0121*** (4.12)
Governance × technical complexity		–0.0049** (–2.06)
Controls	Yes	Yes
Fixed effects	Industry and year	Industry and year
Observations	27,030	27,030
Adjusted *R*^2^	0.4147	0.4111

t-statistics are reported in parentheses. *** *p* < 0.01, ** *p* < 0.05, * *p* < 0.10.

Column (1) shows that organizational learning strengthens the governance-valuation relationship. The interaction coefficient is 0.0049 and significant at the 10 percent level. This pattern is consistent with the idea that learning capability helps firms convert responsible governance principles into operating routines, training, documentation, and cross-functional coordination. Column (2) shows that technical complexity weakens the same relationship. The interaction coefficient is –0.0049 and significant at the 5 percent level. This pattern is consistent with the argument that complex technical systems raise the costs of explainability, auditability, privacy protection, and fairness review.

These moderation estimates use lnTobinQ, so they are interpreted as boundary evidence for external recognition rather than as the primary ROA result. The direction nevertheless supports the theoretical distinction between learning capability and technical complexity. Learning makes responsible governance more implementable. Technical complexity makes governance claims more difficult to verify and operationalize.

The interaction estimates should also be read alongside the heterogeneity splits. The learning interaction suggests that governance information is more strongly recognized when firms disclose stronger learning capability. The technical complexity interaction suggests that the same governance signal is discounted or harder to convert into recognized value when technical systems are more difficult to verify. These patterns are consistent with the proposed implementation logic. They do not show that employees directly experience higher learning climate or technical burden, but they identify firm-level conditions that are plausible for responsible analytics at work.

### 5.5 Robustness, timing, and identification-strengthening evidence

The robustness question is whether the ROA association persists across alternative outcomes, timing structures, dynamic panel estimates, and stronger fixed effects. The first check asks whether the performance pattern is limited to ROA. Table 8 reports an alternative profitability model using return on equity and a lagged-governance valuation model. The ROE coefficient is 0.0026 with a t-statistic of 5.37, which means that a one standard deviation increase in governance corresponds to a 1.15 percentage point higher ROE. This result indicates that the positive operating-performance pattern is not unique to the accounting denominator used in ROA.

**Table 8:** Alternative profitability and lagged valuation checks.

Variable	(1) ROE	(2) lnTobinQ
Responsible governance index	0.0026*** (5.37)	
Lagged responsible governance index		0.0001 (0.16)
Controls	Yes	Yes
Fixed effects	Firm and year	Industry and year
Observations	27,443	23,836
Adjusted *R*^2^	0.0803	0.4163

t-statistics are reported in parentheses. Standard errors are clustered at the firm level. *** *p* < 0.01.

The second column of Table 8 reinforces the secondary status of Tobin's Q. Lagged governance has a coefficient of 0.0001 and a t-statistic of 0.16 in the valuation model. This small estimate suggests that market recognition depends on contemporaneous information processing, disclosure credibility, and investor attention rather than on a stable delayed response to earlier visible governance. The result does not weaken the ROA evidence. It clarifies that the stronger claim concerns operating performance, while valuation remains a more conditional signal.

The ROE check tests whether the profitability result is sensitive to the way organizational performance is scaled. ROA uses assets as the denominator, while ROE uses equity and is more sensitive to leverage. The positive ROE coefficient therefore supports the interpretation that visible governance is associated with operating performance beyond one accounting construction. ROA remains the primary outcome because ROE can be more affected by capital structure. This choice follows the evidence hierarchy established in the Methods section.

The next check addresses performance persistence and dynamic panel bias. Table 9 reports difference GMM estimates with lagged ROA included in the model. Lagged ROA is treated as predetermined and instrumented with its first lag in the differenced equation, while the governance index and controls enter as standard instruments; the estimation uses 24 instruments for 3,215 firms without collapsing, so the instrument count remains far below the number of cross-sectional units. The coefficient on lagged ROA is 0.1824, confirming persistence in operating performance. The governance coefficient is 0.0007 with a z-statistic of 3.23, consistent in direction and magnitude with the baseline fixed-effects estimate of 0.0011. The diagnostic tests support the specification: AR(1) rejects the null as expected after differencing, AR(2) has a *p*-value of 0.787, and the Hansen overidentification test has a *p*-value of 0.078. These diagnostics do not prove exogeneity, but they reduce concern that the baseline result is an artifact of dynamic performance persistence.

**Table 9:** Dynamic panel check using difference GMM.

Variable/diagnostic	(1) ROA
Lagged ROA	0.1824*** (4.32)
Responsible governance index	0.0007*** (3.23)
Controls	Yes
Number of instruments	24
Number of firms	3,215
AR(1) *p*-value	0.000
AR(2) *p*-value	0.787
Hansen *p*-value	0.078
Observations	23,128

z-statistics are reported in parentheses. The model is estimated with one-step difference GMM. Lagged ROA is instrumented with its first lag in the differenced equation; the governance index and controls enter as standard instruments; instruments are not collapsed. *** *p* < 0.01.

The timing evidence examines whether the observed association is compatible with a workplace-governance process rather than simple reverse timing. Table 10 reports lagged governance, lead-placebo, and distributed-lag models. Lagged governance predicts current ROA with a coefficient of 0.0008 and a t-statistic of 4.12. The corresponding lagged valuation coefficient is small and insignificant. Future governance does not predict current ROA or current Tobin's Q, with coefficients of 0.0002 and 0.0004. This lead-placebo pattern is useful because it reduces concern that current performance mechanically anticipates future governance disclosure.

**Table 10:** Timing tests, lead placebo, and distributed lags.

Variable/specification	(1) ROA_*t*_	(2) TobinQ_*t*_	(3) ROA_*t*_	(4) TobinQ_*t*_	(5) ROA_*t*_	(6) TobinQ_*t*_
Panel A: Lagged governance						
Governance_*t*−1_	0.0008*** (4.12)	0.0003 (0.38)			0.0005** (2.21)	0.0001 (0.12)
Panel B: Lead placebo						
Governance_*t*+1_			0.0002	0.0004		
			(0.87)	(0.52)		
Panel C: Distributed lags						
Governance_*t*_					0.0009***	0.0013*
					(4.56)	(1.68)
Controls	Yes	Yes	Yes	Yes	Yes	Yes
Fixed effects	Firm and year	Industry and year	Firm and year	Industry and year	Firm and year	Industry and year
Observations	23,412	22,987	22,156	21,843	21,876	21,534
Adjusted *R*^2^	0.0912	0.4087	0.0876	0.4092	0.0934	0.4098

t-statistics are reported in parentheses. Standard errors are clustered at the firm level. *** *p* < 0.01, ** *p* < 0.05, * *p* < 0.10.

The distributed-lag estimates in Table 10 provide a more nuanced pattern. Current governance remains positively associated with current ROA, and lagged governance also retains a smaller positive coefficient. The valuation model shows a marginal contemporaneous coefficient and no meaningful lagged coefficient. This sequence fits the theoretical argument that visible governance is more closely related to operating coordination and verification burden than to durable market recognition. It also supports cautious wording. The evidence is temporally ordered in a way that is consistent with the proposed association, but the design still cannot rule out all time-varying confounders.

The timing pattern is especially relevant for the organizational psychology interpretation. If governance practices help coordination, documentation, and review routines, the operating association should appear in periods when those routines are visible and in adjacent periods when they remain embedded in work processes. The distributed-lag ROA results fit that expectation. Market recognition is different because investors may react only when governance information is salient, credible, or connected to other performance signals. The weaker Tobin's Q timing evidence therefore supports the decision to treat valuation as secondary.

The final robustness check asks whether common regional or industry shocks explain the baseline coefficient. Table 11 reports strengthened fixed effects. The ROA coefficient remains positive under the baseline year structure, province-by-year fixed effects, and industry-by-year fixed effects. The coefficients are 0.0010, 0.0009, and 0.0011, all significant at the 1 percent level. The valuation coefficients remain positive and marginally significant under province-by-year and industry-by-year structures. These results reduce the likelihood that broad regional policies or industry cycles alone generate the main ROA association.

**Table 11:** Strengthened fixed effects specifications.

Variable/specification	(1) ROA	(2) ROA	(3) ROA	(4) lnTobinQ	(5) lnTobinQ
Responsible governance index	0.0010*** (5.23)	0.0009*** (4.87)	0.0011*** (5.76)	0.0012* (1.72)	0.0014* (1.89)
Firm FE	Yes	Yes	Yes	Yes	Yes
Year FE	Yes	No	No	No	No
Province by year FE	No	Yes	No	Yes	No
Industry by year FE	No	No	Yes	No	Yes
Controls	Yes	Yes	Yes	Yes	Yes
Observations	27,547	27,234	27,312	26,876	26,943
Adjusted *R*^2^	0.0949	0.1023	0.1087	0.4156	0.4234

t-statistics are reported in parentheses. Standard errors are clustered at the firm level. Province by year fixed effects control for province-specific time-varying shocks. Industry by year fixed effects control for industry-specific cyclical patterns. *** *p* < 0.01, * *p* < 0.10.

Taken together, the robustness tables sharpen the evidence boundary. The primary ROA association appears in the baseline model, survives an alternative profitability outcome, remains present in a dynamic panel specification, follows a timing pattern that is inconsistent with the simple lead-placebo concern, and persists under stronger fixed effects. The evidence still remains associational because the design lacks an exogenous governance shock or randomized implementation. The strengthened result sequence therefore supports a bounded organizational psychology claim: visible responsible workplace data governance is reliably associated with downstream organizational performance in this listed-firm setting, while the psychological mechanisms and causal direction require direct employee-level and quasi-experimental evidence.

Two further checks address the time alignment between the cross-sectional survey weights and the panel. First, the index was reconstructed under alternative weight vectors: equal dimension weights and five leave-one-dimension-out variants. Table 12 shows that the ROA coefficient remains positive and significant at the 1 percent level in every variant, from 0.0010 (*t* = 4.70) under equal weights to 0.0007 (*t* = 2.80) in the weakest case, which excludes the highest-weighted dimension of technical trustworthiness. The baseline estimate therefore does not depend on the specific importance structure elicited from respondents. Second, restricting the sample to 2015–2023, the period in which data-intensive technologies diffused most widely across Chinese listed firms, leaves the conclusions unchanged: the governance index remains positively associated with ROA (b = 0.0008, *t* = 3.56, *N* = 20,680) and with lnTobinQ (b = 0.0028, *t* = 3.32, *N* = 20,344). The recent-period ROA coefficient is somewhat smaller than the full-sample estimate, which is consistent with visible governance practices becoming more widespread and therefore less differentiating in later years.

**Table 12:** Time-alignment checks: weight perturbation and recent-period subsample.

Panel A: Weight perturbation, ROA, full sample (N = 27,547)		
Weighting scheme	Coefficient	t-statistic
Baseline survey weights	0.0011***	(6.13)
Equal dimension weights	0.0010***	(4.70)
Excluding organizational governance	0.0009***	(4.08)
Excluding technical trustworthiness	0.0007***	(2.80)
Excluding rights protection	0.0010***	(4.88)
Excluding social impact	0.0011***	(5.62)
Excluding operational mechanisms	0.0010***	(5.14)
**Panel B: 2015–2023 subsample**		
	**(1) ROA**	**(2) lnTobinQ**
Responsible governance index	0.0008***	0.0028***
	(3.56)	(3.32)
Fixed effects	Firm and year	Industry and year
Observations	20,680	20,344
Adjusted *R*^2^	0.125	0.414

t-statistics are reported in parentheses. Standard errors are clustered at the firm level. In Panel A, each row is a separate regression with firm and year fixed effects and the full control vector; the index is reconstructed under the stated weighting scheme before estimation. *** *p* < 0.01.

### 5.6 Ownership and implementation heterogeneity

The heterogeneity question is whether the governance-performance association differs across organizational contexts. Table 13 reports ownership and implementation split-sample evidence. The ownership split shows a near-zero coefficient among state-owned enterprises and a positive coefficient among non-state-owned enterprises. The SOE coefficient is 0.0000 with a t-statistic of 0.14, while the non-SOE coefficient is 0.0014 with a t-statistic of 5.76. The Chow test rejects equality across the two groups with *F* = 18.76. This pattern suggests that responsible governance is more strongly associated with ROA where firms face stronger market discipline and may have stronger incentives to implement governance practices substantively.

**Table 13:** Ownership and implementation heterogeneity.

Variable/statistic	(1)	(2)	(3)	(4)	(5)
	SOE	Non-SOE	High Tech	Low Tech	High Learn
	ROA	ROA	ROA	ROA	ROA
Responsible governance index	0.0000 (0.14)	0.0014*** (5.76)	0.0008*** (3.12)	0.0013*** (5.23)	0.0015*** (5.87)
Controls	Yes	Yes	Yes	Yes	Yes
Fixed effects	Firm and year	Firm and year	Firm and year	Firm and year	Firm and year
Observations	8,852	18,695	13,654	13,893	13,512
Adjusted *R*^2^	0.0521	0.1132	0.0876	0.1023	0.1156
Chow test, SOE vs. non-SOE	*F* = 18.76***				
Chow test, high vs. low tech			*F* = 4.23**		

t-statistics are reported in parentheses. High and low technical complexity are split at the median of Tech_Complexity. The high-learning subsample includes firms above the median of Org_Learning. *** *p* < 0.01, ** *p* < 0.05.

The implementation-condition splits align with the moderation evidence. Firms below the median of technical complexity have a larger governance coefficient of 0.0013, while firms above the median have a smaller coefficient of 0.0008. The Chow test for the high-vs.-low technical-complexity split is significant at the 5 percent level. Firms above the median of organizational learning have a coefficient of 0.0015, larger than the full-sample baseline. These patterns preserve the source heterogeneity evidence while keeping ownership as contextual rather than central to the Frontiers argument.

## 6 Discussion

### 6.1 Main findings in organizational psychology terms

The evidence indicates that visible responsible workplace data governance is positively associated with downstream organizational performance among Chinese listed firms. The primary result is the ROA association, which remains stable across baseline, alternative profitability, dynamic panel, timing, and strengthened fixed effects specifications. The finding is best interpreted as organizational evidence that firms disclosing more transparent, accountable, privacy-respecting, and auditable data governance practices also show better operating performance after major controls are included. The result does not prove that governance changes individual employee trust or fairness perceptions because the empirical design does not measure those states directly.

The contribution to organizational psychology is threefold. First, the study supplies a measurement bridge: an employee-weighted, disclosure-based index that lets fairness-relevant and trust-relevant governance constructs travel into large firm-year panels. Second, it keeps the levels of analysis separate and explicit, so firm-level associations are not mistaken for individual-level psychological evidence. Third, it identifies implementation boundaries, learning capability and technical complexity, that individual-level studies of algorithmic work rarely observe.

The organizational psychology interpretation rests on the content of the governance measure and the theory linking data practices to workplace behavior. The index covers technical trustworthiness, organizational governance, rights protection, social impact, and operational mechanisms. These dimensions correspond to conditions that shape trust-relevant transparency, procedural fairness, voice, human oversight, and coordination in data-intensive work. Prior workplace and AI research explains why these conditions matter for employee and managerial responses to monitoring, algorithmic control, and human-AI collaboration. The firm-year estimates then show that visible governance practices are associated with downstream organizational outcomes in a large listed-firm setting.

This interpretation contributes to organizational psychology by connecting micro-relevant constructs to firm-year evidence without collapsing the levels of analysis. Trust, perceived fairness, and voice are not directly measured, but they guide the theoretical meaning of the governance dimensions. ROA and Tobin's Q are not psychological outcomes, but they show whether visible governance practices are associated with organizational consequences. The contribution lies in building a measurable bridge between responsible analytics at work and organizational performance while stating which part of the bridge remains theoretical and which part is estimated.

The expanded evidence sequence also clarifies how firm-level and employee-level research can be joined. The questionnaire evidence identifies which governance dimensions employees and workplace professionals judge as salient. Annual-report scoring then tracks the visible presence of those dimensions across firm-years. The regression evidence shows whether this visible governance layer is associated with organizational outcomes. Future studies can use the same measurement structure to test whether employees in higher-scoring firms actually report stronger procedural fairness, trust in analytics, voice, or psychological safety. This step is necessary because the present evidence establishes an organizational association, while the individual-level processes remain theoretically grounded.

### 6.2 Transparency, accountability, trust, and fairness

The findings speak most directly to transparency and accountability as organizational design conditions. Responsible workplace data governance creates visible procedures for how firms define responsibilities, protect rights, manage risks, audit algorithms, and document data use. These procedures can make workplace data systems easier to understand and challenge. In organizations where employees and managers rely on analytics and automated recommendations, such procedures can reduce ambiguity about who is responsible for decisions and how data-based outputs are reviewed. That clarity is relevant to trust and perceived fairness.

The compliance-cost pathway provides one measurable sign of this coordination logic. Higher governance scores are associated with lower compliance cost, and lower compliance cost is associated with higher ROA. The mediation proportion is modest, which is theoretically plausible. Administrative expense captures only part of the friction created by verification, documentation, and coordination. Trust-relevant and fairness-relevant processes may operate through other pathways that are not captured by the available firm-year data. The mechanism evidence should therefore be used as partial support for the governance-friction argument, not as proof of a complete psychological pathway.

The same caution applies to disclosure visibility. A firm may disclose governance practices because it has invested in routines, because it wants to signal responsibility, or because regulation and external audiences pressure it to report. These motives can coexist. The findings remain useful because visible practices are part of the workplace governance environment, but future research should test whether visible disclosure corresponds to employee perceptions of transparency, fairness, and trust inside the organization.

The timing and robustness evidence strengthen this bounded interpretation. Lead-placebo estimates do not show that future governance predicts current outcomes, and strengthened fixed effects retain the positive ROA association after regional or industry-year shocks are absorbed. These patterns make the transparency and accountability interpretation more credible than a simple static-disclosure explanation. They still leave open the possibility that better managers both disclose more governance practices and operate more effective workplaces. Responsible workplace data governance is therefore treated as an observable organizational capability associated with performance, not as a proven cause of employee trust or fairness.

### 6.3 Learning, technical complexity, and implementation

The boundary evidence indicates that implementation capacity matters. Organizational learning strengthens the governance relationship because responsible data practices require more than written rules. Firms must train employees, update procedures, coordinate across departments, and adapt to new technical and regulatory requirements. A learning-capable firm can translate governance principles into routines that employees and managers can use in daily work. This result connects responsible analytics governance with the organizational psychology literature on learning, adaptation, and technology-mediated coordination.

Technical complexity weakens the governance relationship because complex systems are more difficult to explain, audit, and control. When data architectures involve many layers, proprietary algorithms, or intensive R&D processes, employees and external audiences may find it harder to evaluate whether governance claims are credible. Firms may also spend more resources documenting systems, validating decisions, and managing privacy or fairness risks. The negative interaction and the high-vs.-low technical complexity split point to the same implementation constraint. Responsible governance may require larger investments in explainability, auditability, and documentation when technical complexity is high.

The heterogeneity results add organizational context to the learning and complexity findings. The stronger association among non-state-owned enterprises suggests that governance routines may matter more when firms face sharper market incentives and weaker administrative protection. The larger coefficient among low-complexity firms suggests that governance is easier to convert into operating benefits when systems are more interpretable and less costly to verify. The high-learning split shows the opposite enabling condition: organizations with stronger learning signals appear better positioned to absorb and apply governance routines. These patterns indicate that responsible governance should be studied together with implementation capacity, incentive structure, and technical architecture.

### 6.4 Practical implications

Managers can treat responsible workplace data governance as a work-system capability. The findings suggest that governance practices are more useful when they are embedded in routines that employees and managers can understand and apply. Practical steps include cross-functional governance teams, documented data-use rules, employee-facing explanations of data collection and automated decision processes, appeal channels, privacy review, fairness testing, and accountable audit trails. These practices should be linked to training and learning routines so that governance is not confined to compliance documents.

The evidence also suggests that firms should match governance design to technical complexity. Organizations with complex data systems need stronger documentation, interpretable model design, human review points, and internal audit capacity because the cost of verification is higher. Firms with lower learning capability may need staged implementation, training, and external advisory support before governance routines can improve coordination. The results do not justify universal claims that every governance investment produces immediate performance gains. They indicate that visible governance is associated with better organizational outcomes under specific implementation conditions.

The Chinese setting shapes how these findings travel. The sample period covers the fastest corporate digitalization episode among large economies and a rapid sequence of data regulation, including the Cybersecurity Law of 2017, the Data Security Law of 2021, and the Personal Information Protection Law of 2021. Visible data governance in this setting therefore reflects both voluntary organizational design and escalating regulatory expectations, and the stronger associations among non-state-owned firms suggest that market discipline and compliance pressure interact rather than substitute. This institutional trajectory makes Chinese listed firms a leading case for studying how workplace data governance becomes institutionalized under regulatory acceleration, and it defines a comparative agenda: whether the governance-performance association replicates in jurisdictions where data regulation arrived earlier or remains more fragmented, and whether regulatory-period heterogeneity within China itself conditions the association.

### 6.5 Limitations and future research

The first limitation is measurement. The governance index is based on annual-report visible practices. It cannot fully distinguish substantive routines from strategic disclosure. It also cannot observe informal practices, employee experiences, or hidden data-processing procedures. Future research should triangulate disclosure-based measures with audits, regulatory enforcement records, internal policy documents, employee surveys, and behavioral indicators of workplace data governance.

A further measurement limitation concerns the mediator. Administrative expense over revenue is a coarse proxy for governance friction: it aggregates restructuring, managerial expansion, and other overhead with the verification and coordination costs the theory targets, and it cannot isolate data-governance-specific friction from general administrative efficiency. The 10.8 percent mediation share should therefore be read as an upper-bound signal that a friction pathway is compatible with the data, not as an estimate of the pathway's true size. Direct measures of verification effort, such as audit hours, documentation workload, or dispute frequency, would allow sharper mediation tests.

The moderator proxies are similarly distant from their constructs. Keyword frequencies capture learning-related disclosure rather than realized learning capability, and the technical-complexity composite blends firm research intensity with city-level digitization rather than measuring the auditability burden of specific systems. Direct measures of training participation, routine updating, and system explainability would sharpen both boundary tests.

The second limitation is the absence of direct psychological outcomes. The survey provides employee importance ratings for governance dimensions and indicators, but it does not measure trust, perceived fairness, wellbeing, voice, psychological safety, or learning climate. Trust and fairness are therefore interpreted as theory-grounded mechanisms, not as directly estimated mediators. Future studies should link firm-level governance measures to employee-level responses in multilevel designs.

The third limitation is identification. Fixed effects, difference GMM, timing tests, lead-placebo tests, and strengthened fixed effects improve credibility, but they do not remove every possible time-varying confound. Better-performing firms may still be more able to invest in governance, and unobserved managerial quality may change over time. Future research could exploit regulatory shocks, staggered adoption, third-party audit events, or internal governance reforms to strengthen causal inference.

The fourth limitation is scope. The setting is Chinese A-share non-financial listed firms from 2011 to 2023. Listed firms face disclosure incentives and regulatory expectations that differ from those of unlisted firms and firms in other jurisdictions. The observed relationships may also vary across industries, legal systems, and cultural expectations about employer data use. Future research should compare countries and sectors, examine regulatory-period heterogeneity, and test whether responsible workplace data governance produces spillovers across supply chains, platforms, and collaborative work networks.

## 7 Conclusion

Responsible workplace data governance is an organizational psychology object of study, not merely a compliance topic, because data-intensive work depends on whether employees and managers can understand, question, and trust the rules governing data collection, automated processing, privacy protection, human oversight, and accountability. This study develops an employee-informed measure of visible responsible workplace data governance and applies it to Chinese A-share non-financial listed firms from 2011 to 2023. The measure uses 519 listed-company employee questionnaires to weight five dimensions and 19 indicators, then scores annual-report evidence to construct a firm-year governance index.

The empirical evidence shows that higher visible governance is positively associated with ROA, the primary downstream organizational performance outcome. A one standard deviation increase in the index corresponds to a 0.49 percentage point higher ROA. Tobin's Q evidence is positive but weaker. The mechanism evidence supports a partial governance-friction pathway through lower compliance cost, and the moderation evidence shows that organizational learning strengthens while technical complexity weakens the governance relationship. Ownership heterogeneity indicates stronger associations among non-state-owned enterprises.

The contribution is a measurement and evidence bridge between responsible analytics at work and organizational performance. The study does not claim that annual reports directly observe trust, fairness, voice, wellbeing, or learning climate. It shows that employee-weighted, disclosure-visible governance practices are associated with organizational outcomes in ways that are consistent with transparency, accountability, fairness, learning, and coordination mechanisms. Future work should connect this firm-level evidence to direct employee-level psychological data and stronger causal designs.

For Frontiers readers, the main implication is measurement discipline. Workplace data governance can be studied as an observable organizational capability only when the construct is separated from broad technology enthusiasm and from unverified claims about employee experience. The index developed here offers one scalable route: it combines employee-informed weights with public evidence on governance routines, technical safeguards, rights protection, social impact, and operational accountability. The expanded main-text evidence shows that the association is visible across baseline, robustness, dynamic, timing, fixed-effects, and heterogeneity tests while remaining bounded by disclosure measurement and observational design. The evidence should therefore be read as a firm-level starting point for organizational psychology research on data-intensive work. It identifies where responsible governance is visible, where it appears linked to downstream performance, and where individual-level psychological mechanisms still need direct measurement.

## Data Availability

The datasets analyzed for this study are available from the corresponding author upon reasonable request, subject to restrictions imposed by the original data providers and the confidentiality commitments made to survey participants.

## References

[B1] Ang J. S. Cole R. A. Lin J. W. (2000). Agency costs and ownership structure. J. Finan. 55, 81–106. doi: 10.1111/0022-1082.00201

[B2] Awan U. Ahmed S. Albishri N. Kalisz D. Szabo-Szentgroti G. (2026). Organisational responsible ai implementation and organisational foresight: the role of leadership control and managerial cognitive flexibility. Technol. Forecast. Soc. Change 227:124623. doi: 10.1016/j.techfore.2026.124623

[B3] Berretta S. Tausch A. Peifer C. Kluge A. (2023). The job perception inventory: considering human factors and needs in the design of human-ai work. Front. Psychol. 14:1128945. doi: 10.3389/fpsyg.2023.1128945 37287772 PMC10243195

[B4] Calacci D. Stein J. (2023). From access to understanding: collective data governance for workers. Eur. Labour Law J. 14, 253–282. doi: 10.1177/20319525231167981

[B5] Chen N. Zhang X. (2025). When misunderstanding meets artificial intelligence: the critical role of trust in human-ai and human-human team communication and performance. Front. Psychol. 16:1637339. doi: 10.3389/fpsyg.2025.1637339 41209806 PMC12589011

[B6] Collins P. Atkinson J. (2023). Worker voice and algorithmic management in post-brexit britain. Transfer-Eur. Rev. Labour Res. 29, 37–52. doi: 10.1177/10242589221143068

[B7] Colquitt J. A. Conlon D. E. Wesson M. J. Porter C. O. L. H. Ng K. Y. (2001). Justice at the millennium: a meta-analytic review of 25 years of organizational justice research. J. Appl. Psychol. 86, 425–445. doi: 10.1037/0021-9010.86.3.425 11419803

[B8] de Menezes L. M. Escrig A. B. (2019). Managing performance in quality management a two-level study of employee perceptions and workplace performance. Int. J. Operat. Prod. Manag. 39, 1226–1259. doi: 10.1108/IJOPM-03-2019-0207

[B9] de Menezes L. M. Escrig-Tena A. B. (2023). Performance measurement systems in the health and care sector: are targets and monitoring additional demands or resources for employees? Int. J. Operat. Prod. Manag. 43, 302–329. doi: 10.1108/IJOPM-12-2022-0763

[B10] Ebert I. Wildhaber I. Adams-Prassl J. (2021). Big data in the workplace: privacy due diligence as a human rights-based approach to employee privacy protection. Big Data Soc. 8:3051. doi: 10.1177/20539517211013051

[B11] Friede G. Busch T. Bassen A. (2015). ESG and financial performance: aggregated evidence from more than 2000 empirical studies. J. Sustain. Finan. Invest. 5, 210–233. doi: 10.1080/20430795.2015.1118917

[B12] Gagne M. Parent-Rocheleau X. Bujold A. Gaudet M.-C. Lirio P. (2022). How algorithmic management influences worker motivation: a self-determination theory perspective. Can. Psychol. 63, 247–260. doi: 10.1037/cap0000324

[B13] Gal U. Jensen T. B. Stein M.-K. (2020). Breaking the vicious cycle of algorithmic management: a virtue ethics approach to people analytics. Inform. Organiz. 30:100301. doi: 10.1016/j.infoandorg.2020.100301

[B14] Gao X. Wu W. Guo Y. Lu L. (2025). Promotion or suppression? Effect of perceived algorithmic control on service performance of gig workers. Soc. Behav. Pers. 53:14812. doi: 10.2224/sbp.14812

[B15] Gaudio G. (2024). Litigating the algorithmic boss in the eu: a (legally) feasible and (strategically) attractive option for trade unions? Int. J. Comp. Labour Law Indus. Relat. 40, 91–130. doi: 10.54648/IJCL2024002

[B16] Gompers P. Ishii J. Metrick A. (2003). Corporate governance and equity prices. Quart. J. Econ. 118, 107–155. doi: 10.1162/00335530360535162

[B17] Hiremath S. P R. Konek S. S. Bhavikatti V. I. Vemula R. B O. (2025). Artificial intelligence (ai) governance in organizational decision-making: balancing autonomy, accountability and transparency. J. Entrepren. Public Policy. 1–24. doi: 10.1108/JEPP-04-2025-0112

[B18] Huo W. Wang Y. Liang B. Song M. Xie J. (2026). Algorithmic control in app-work platforms: exploring its curvilinear impact on work well-being. Eur. Manag. Rev. 23, 3–21. doi: 10.1111/emre.70005

[B19] Jarrahi M. H. Newlands G. Lee M. K. Wolf C. T. Kinder E. Sutherland W. (2021). Algorithmic management in a work context. Big Data Soc. 8:20539517211020332. doi: 10.1177/20539517211020332

[B20] Jones C. H. Rosemarino N. Dolsten C. R. (2025). People analytics and organizational psychology: building a high-performance, innovative culture in the pharmaceutical industry. Drug Discov. Today 30:104476. doi: 10.1016/j.drudis.2025.104476 40947046

[B21] Lang J. J. Yang L. F. Cheng C. Cheng X. Y. Chen F. Y. (2023). Are algorithmically controlled gig workers deeply burned out? An empirical study on employee work engagement. BMC Psychol. 11:354. doi: 10.1186/s40359-023-01402-0 37876010 PMC10598991

[B22] Li Y. Zhao L. Cao C. Yang D. (2025). The double-edged sword effect of algorithmic transparency: an empirical study of gig workers' work disengagement under algorithmic management. Inform. Manag. 62:104100. doi: 10.1016/j.im.2025.104100

[B23] Liang T. Zhang Y. Xiang D. Zhu L. (2026). Voice in the algorithmic era: how perceived algorithmic control influences gig workers' voice behavior. Front. Psychol. 16:1637658. doi: 10.3389/fpsyg.2025.1637658 41602665 PMC12833266

[B24] Liang X. Fu W. Luo P. Huo Y. (2024). Challenge or hindrance? The dual impact of algorithmic control on gig workers' prosocial service behaviors. Behav. Sci. 14:497. doi: 10.3390/bs14060497 38920830 PMC11200372

[B25] Lin Q. Sun R. Zhu Q. (2025). Perceived algorithmic control and gig workers' work engagement: assessing the mediating role of psychological empowerment and the moderating effect of deep acting. BMC Psychol. 13:1237. doi: 10.1186/s40359-025-03570-7 41204295 PMC12595896

[B26] Lind E. A. van den Bos K. (2002). When fairness works: toward a general theory of uncertainty management. Res. Organiz. Behav. 24, 181–223. doi: 10.1016/S0191-3085(02)24006-X

[B27] Liu S. Wang Y. Xu G. Zhao W. (2026). Navigating the light and dark sides of algorithmic control: a double-sided impact on gig worker well-being. Employee Relat. 48, 572–595. doi: 10.1108/ER-07-2025-0583

[B28] Liu Y. Li Y. (2025). Does human-ai collaboration promote or hinder employees' safety performance? A job demands-resources perspective. Saf. Sci. 188:106872. doi: 10.1016/j.ssci.2025.106872

[B29] Madanchian M. Taherdoost H. (2025). Ethical theories, governance models, and strategic frameworks for responsible ai adoption and organizational success. Front. Artif. Intell. 8:1619029. doi: 10.3389/frai.2025.1619029 40741285 PMC12307427

[B30] Mbare B. Perkio M. Koivusalo M. (2024). Algorithmic management, wellbeing and platform work: understanding the psychosocial risks and experiences of food couriers in finland. Labour Industry 34, 386–411. doi: 10.1080/10301763.2024.2423442

[B31] McNall L. A. Roch S. G. (2009). A social exchange model of employee reactions to electronic performance monitoring. Hum. Perform. 22, 204–224. doi: 10.1080/08959280902970385

[B32] Migliora G. (2026). Platform work and algorithmic management: protecting workers irrespectively of their employment status. Comput. Law Sec. Rev. 60:106267. doi: 10.1016/j.clsr.2026.106267

[B33] Munz P. Hennick M. Stewart J. (2023). Maximizing ai reliability through anticipatory thinking and model risk audits. AI Magaz. 44, 173–184. doi: 10.1002/aaai.12099

[B34] Pawirosumarto S. Kurniawan B. (2026). Datafied selves at work: ethical boundaries of surveillance in people analytics. J. Business Ethics. doi: 10.1007/s10551-026-06272-1

[B35] Pesole A. (2025). Algorithmic management and the platformisation of work in europe: evidence from spain and germany. Indian J. Labour Econ. 68, 367–394. doi: 10.1007/s41027-024-00544-y

[B36] Shamim S. Yang Y. Ul Zia N. Khan Z. Shariq S. M. (2023). Mechanisms of cognitive trust development in artificial intelligence among front line employees: an empirical examination from a developing economy. J. Bus. Res. 167:114168. doi: 10.1016/j.jbusres.2023.114168

[B37] Singh M. Davidson W. N. (2003). Agency costs, ownership structure and corporate governance mechanisms. J. Banking Finan. 27, 793–816. doi: 10.1016/S0378-4266(01)00260-6

[B38] Tuomi A. Jianu B. Hua M. Roelofsen M. Ascencao M. P. (2024). Strategies for communicating and mitigating algorithmic control on delivery platforms. Convergence 30, 1685–1709. doi: 10.1177/13548565241270908

[B39] Wang B. Liu S. Luo C. (2026). How does human-ai collaboration task complexity affect employee work engagement? *the roles of humble leadership and ai self-efficacy. Frontiers In Psychology* 17. doi: 10.3389/fpsyg.2026.1767967 41743435 PMC12929112

[B40] Wang Y. Wang Z. Li J. (2024). Does algorithmic control facilitate platform workers' deviant behavior toward customers? The ego depletion perspective. Comput. Hum. Behav. 156:108242. doi: 10.1016/j.chb.2024.108242

[B41] Won J. Lee D. Lee J. (2023). Understanding experiences of food-delivery-platform workers under algorithmic management using topic modeling. Technol. Forecast. Soc. Change 190:122369. doi: 10.1016/j.techfore.2023.122369

[B42] Wu F. Hu H. Lin H. Ren X. (2021). Enterprise digital transformation and capital market performance: empirical evidence from stock liquidity. Manag. World 37, 130–144. doi: 10.19744/j.cnki.11-1235/f.2021.0097

[B43] Zhang L.-X. Li J.-M. Liu K.-X. Zhang D. (2025). How does algorithmic control affect the work engagement of gig workers? The role of perceived algorithmic fairness and psychological contract. Asia Pac. J. Hum. Resour. 64:70045. doi: 10.1111/1744-7941.70045

[B44] Zhao H. Yuan B. Gong Y. Liao Y. (2026). Platform algorithmic control and work outcomes of food delivery employees. Serv. Indus. J. 46, 369–415. doi: 10.1080/02642069.2025.2477454

[B45] Zhao Y. Ren F. Fan M. (2025). The effect of developmental electronic performance monitoring on employee innovative behavior. Sci. Rep. 15:32005. doi: 10.1038/s41598-025-12326-7 40885753 PMC12398480

