# Supplementary material for: Responsible workplace data governance and organizational performance: evidence from Chinese listed firms
Source: Front Psychol. 2026 Aug 12;17:1891689. doi: 10.3389/fpsyg.2026.1891689 (PMC13506833; doi:10.3389/fpsyg.2026.1891689)
Supplement: Supplementary file 1 [file Data_Sheet_1.pdf]

Supplementary Material

1 SUPPLEMENTARY MATERIAL

This file provides supplementary material for the article "Responsible Workplace Data Governance and Organizational Performance: Evidence from Chinese Listed Firms." It reports the indicator framework, employee weighting evidence, variable definitions, empirical strategy details, additional descriptive evidence, robustness tests, heterogeneity analysis, survey calibration evidence, and extended practical implications. The material preserves the empirical result families used in the article while using the Frontiers supplementary-material format.

1.1 Responsible Workplace Data Governance Indicator Framework and Scoring Evidence Rules

The responsible workplace data governance index is organized around five first-level dimensions and nineteen second-level indicators. The framework separates organizational foundations, technical system properties, data-subject rights, social externalities, and operational enforcement mechanisms because each dimension captures a different observable component of responsible data governance at work. This structure reduces the risk that the index records only broad disclosure volume. It requires evidence of specific routines, responsibilities, controls, and reporting channels. The organizational governance dimension captures the organizational foundation for responsible analytics implementation. Awareness maturity measures the evolution of organizational cognition regarding data governance, from basic recognition to strategic integration. Observable evidence includes committee formation, strategic plan integration, and executive communications. Leadership commitment assesses whether senior executives provide resources and decision-making authority for governance initiatives, with evidence drawn from dedicated budget allocation, board-level oversight, and executive responsibility assignment. Cross-departmental collaboration evaluates coordination mechanisms across technology, legal, customer-operations, and human resources functions. Observable evidence includes cross-functional task forces, shared reporting structures, and joint training programs. Policy completeness examines whether firms establish execution frameworks covering the data lifecycle, including documented policies, procedural guidelines, and compliance checklists.

The technical trustworthiness dimension captures the reliability, security, transparency, and fairness of technical systems. Technical robustness assesses system resistance to attacks and fault tolerance. Privacy and data governance evaluate lifecycle compliance and privacy protection in data handling. Transparency measures decision-process traceability and explanation capacity. Algorithmic fairness examines group bias and output inclusiveness. Technical tools such as explainable artificial intelligence, fairness audits, traceability systems, federated learning, and privacy-preserving computation strengthen verification and reduce uncertainty in data-intensive work. The rights protection dimension focuses on the interests of employees, customers, and other data subjects who can observe governance failures. Human oversight rights ensure that humans retain review and termination authority over high-stakes decisions. User empowerment enables users to manage their data and appeal decision outcomes. Fundamental rights protection prevents privacy violations and unequal treatment. Whistleblower system effectiveness provides protected anonymous reporting channels with rapid response capacity. Observable evidence includes published rights statements, opt-out mechanisms, appeal procedures, and whistleblower hotline disclosures.

The social impact dimension evaluates broader effects of big data applications on society, the environment, and employment. Social welfare measures whether systems promote social equity and community wellbeing. Environmental impact assesses system energy consumption and carbon emissions. Employment impact evaluates job displacement effects and retraining support. Malicious use prevention examines whether firms block criminal misuse technologies such as deepfakes. Corporate disclosures on social impact remain sparse because firms face weaker mandatory reporting requirements for societal externalities than for privacy or security matters. The index accommodates sparse disclosure by assigning scores based on the presence and quality of available evidence. The operational mechanisms dimension captures governance infrastructure that enables closed-loop verification and accountability. Accountability mechanisms ensure responsibility traceability and enforcement. Risk management identifies full-process risks and develops tiered response plans. Ethics compliance auditing verifies algorithmic bias and privacy compliance. Observable evidence includes audit committee reports, third-party certification disclosures, incident response documentation, and remediation tracking. Table S1 reports the resulting indicator framework.

Table S1. Responsible Workplace Data Governance Indicator Framework

First-level dimension	Second-level indicators
Organizational governance	Ethics awareness maturity; leadership commitment; cross-departmental collaboration; policy completeness
Technical trustworthiness	Technical robustness; privacy and data governance; transparency; algorithmic fairness
Rights protection	Human oversight rights; user empowerment; fundamental rights protection; whistleblower system effectiveness
Social impact	Social welfare; environmental impact; employment impact; malicious use prevention
Operational mechanisms	Accountability mechanisms; risk management; ethics compliance auditing

Notes: The framework contains five first-level dimensions and nineteen second-level indicators. Indicator scoring uses annual-report textual evidence, disclosed governance structures, and observable process controls.

1.2 Questionnaire Design and Weight Determination

The weighting procedure combines respondent judgment and information-content adjustment. The survey instrument collected respondent and firm characteristics, direct weight allocation across the five first-level dimensions, five-point Likert ratings for all nineteen second-level indicators, and evaluations of potential text-analysis keywords. After excluding invalid and logically inconsistent responses, the study obtained 519 valid questionnaires from listed-company employees and related professionals. The respondent sample includes 46.24% male respondents, 59.15% respondents aged 31 to 40 years, 80.54% respondents holding bachelor's degrees, and 71.87% respondents in non-managerial professional roles. Data management experience is concentrated in mid-career groups: 40.08% report 3 to 5 years of experience, and 26.97% report 6 to 10 years. Industry coverage includes internet platforms and services at 24.66%, intelligent manufacturing at 24.28%, e-commerce and retail at 15.22%, energy and basic materials at 10.40%, and other sectors.

The final weighting approach integrates Analytic Hierarchy Process judgment with entropy weighting. Respondents allocated the largest mean weight to technical trustworthiness at 25.69%, followed by organizational governance at 21.58%, rights protection at 18.89%, operational mechanisms at 17.09%, and social impact at 16.76%. Within each dimension, indicator scores enter with equal weights. The dimension-level weights combine direct respondent weight allocation with entropy-based information content in equal proportions. Reliability analysis confirms adequate internal consistency because Cronbach's alpha coefficients exceed 0.70 for all dimensions. Within-group agreement is also sufficient for aggregation because the median r_{wg} exceeds 0.70. The final index aggregates weighted dimension scores into a

standardized composite index scaled from 0 to 100. Table S2 reports the dimension-level weights and mean ratings.

Table S2. Dimension Weights and Mean Likert Scores

Dimension	Mean weight (%)	Mean Likert score
Technical trustworthiness	25.69	4.12
Organizational governance	21.58	4.04
Rights protection	18.89	4.04
Operational mechanisms	17.09	4.15
Social impact	16.76	3.95

Notes: Weights are based on 519 valid questionnaires and combine direct respondent allocation with entropy-based information adjustment.

1.3 Data Sources, Sample Screening, and Variable Definitions

The sample construction follows exclusion rules designed to improve comparability across listed firms. The study excludes financial and insurance firms because their regulatory environments, capital structures, and performance metrics differ from those of general corporations. Firms with Special Treatment (ST), ∗ST, or Particular Transfer (PT) designations are excluded because abnormal operating conditions and delisting risk introduce confounding variation. Observations with missing core variables or key controls are dropped. Continuous variables are winsorized at the 1st and 99th percentiles to reduce the influence of extreme values while retaining observations. The scoring procedure maps textual evidence from Management Discussion and Analysis sections to indicator categories using standardized keyword dictionaries and phrase patterns. Indicator scores are aggregated within dimensions, and dimension scores are then aggregated into the responsible workplace data governance index. This text-based design makes the measure replicable across firms and years while preserving the employee-informed weighting structure described above. The moderating variables capture organizational capacity and environmental constraints. Organizational learning capability, denoted Org_Learning, proxies the dynamic capability for absorbing and implementing external governance norms. It is constructed as the standardized frequency of a 50-term learning dictionary in annual-report Management Discussion and Analysis sections; the full term list appears below, and firm-years without usable text enter full-panel models at the standardized mean of zero. Stronger learning capability should help firms translate responsible analytics principles into operational improvements because employees and managers can absorb external standards, adapt internal routines, and update process controls more quickly.

Technical complexity, denoted Tech_Complexity, captures implementation constraints that can weaken responsible governance benefits. It is a composite measure: firm research and development expenditure over operating revenue (in percent) is averaged with the city-level digitization index of the firm's registered location, and the city-level index substitutes when firm research and development intensity is missing. The composite is standardized before entering the moderation models. High technical complexity increases the difficulty and cost of achieving explainability, fairness auditing, privacy protection, and auditability.

Compliance cost, denoted Compliance_Cost, captures the administrative burden associated with regulatory adherence. It is measured as administrative expenses divided by operating revenue. It is a reverse indicator because higher values indicate greater compliance costs. Responsible governance can reduce compliance costs through process optimization, risk prevention, and lower verification frictions. Control variables address potential confounds: firm size captures scale and visibility, leverage controls for capital structure, board size and independent-director ratio capture governance quality, ownership concentration captures agency relationships, and firm age controls for lifecycle effects. Table S3 reports the variable definitions.

Table S3. Variable Definitions

Type	Variable	Symbol	Measurement
Dependent	Downstream organizational performance	ROA	Net income divided by average total assets
Dependent	External market recognition	lnTobinQ	Natural logarithm of (Tobin's Q + 1); Tobin's Q is market value divided by asset replacement cost
Independent	Responsible workplace data governance	Ethics_Index	Composite index from questionnaire and AHP-entropy weighting, scaled 0 to 100
Moderator	Organizational learning	Org_Learning	Frequency of a 50-term learning dictionary per 10,000 MD&A words, z-standardized; missing text coverage imputed at the standardized mean of zero
Moderator	Technical complexity	Tech_Complexity	Mean of firm R&D expenditure over operating revenue (percent) and the city-level digitization index; the city index substitutes when firm R&D is missing; standardized in moderation models
Mediator	Compliance cost	Compliance_Cost	Administrative expenses divided by operating revenue, reverse indicator
Control	Firm size	Size	Natural logarithm of total assets
Control	Leverage	Leverage	Total liabilities divided by total assets
Control	Board size	Board_Size	Natural logarithm of board member count
Control	Independent directors	Indep_Director	Independent directors divided by board size
Control	Ownership concentration	Top1	Largest shareholder ownership percentage
Control	Firm age	Age	Natural logarithm of listing years
Control	Financing constraints	SA_index	Hadlock-Pierce size-age index
Control	Industry concentration	HHI	Revenue-based Herfindahl-Hirschman index within CSMAR industry
Control	Digital transformation intensity	Digital_transformation	Text-mined digital keyword frequency in the annual report, following Wu et al. (2021)

Notes: MD&A denotes Management Discussion and Analysis. Continuous variables are winsorized at the 1st and 99th percentiles.

1.4 Empirical Strategy Technical Details

The moderation tests examine whether organizational learning capability and technical complexity condition the association between responsible workplace data governance and performance. The estimating equation is:

$$\begin{aligned} Performance_{it} &= \beta_0 + \beta_1 Ethics\_Index_{it} + \beta_2 Moderator_{it} \\ &\quad + \beta_3 (Ethics\_Index_{it} \times Moderator_{it}) + \sum_j \beta_j Controls_{it} + \mu_i + \lambda_t + \varepsilon_{it}. \end{aligned} \tag{S1}$$

A positive interaction coefficient for organizational learning indicates that learning capability amplifies the performance association of responsible governance. A negative interaction coefficient for technical complexity indicates that complexity weakens this association.

The mediation tests examine whether compliance cost reduction carries part of the association between responsible governance and firm performance. The analysis follows the Baron and Kenny stepwise approach and supplements it with bootstrap confidence intervals. First, the study regresses performance on responsible governance to estimate the total association. Second, the study regresses compliance cost on responsible governance to assess path a. Third, the study regresses performance on responsible governance and compliance cost to estimate the direct association, denoted path c', and the compliance-cost coefficient, denoted path b. The evidence is consistent with partial mediation when responsible governance predicts compliance cost, compliance cost predicts performance conditional on governance, and the direct association declines after including compliance cost. Bootstrap resampling with 5,000 iterations provides confidence intervals for the indirect effect. This design evaluates whether the data fit the hypothesized mediation structure. The design remains associational because compliance cost is observed and is not randomly assigned.

1.5 Additional Descriptive Evidence

Table S4 reports pairwise Pearson correlations. The governance index correlates positively with ROA ($r = 0.159$, $p < 0.01$) and negatively with compliance cost ($r = -0.166$, $p < 0.01$). These correlations provide preliminary evidence consistent with the downstream performance and governance-friction hypotheses. The governance index correlates negatively with $\ln TobinQ$ in the raw data ($r = -0.092$, $p < 0.01$), while the multivariate regressions report positive conditional coefficients for valuation. The sign change reflects confounding by firm characteristics, especially firm size. Larger firms tend to have higher governance scores and lower valuation multiples, creating a negative unconditional association. Fixed effects and controls isolate the conditional relationship between governance and valuation. Correlations among explanatory variables remain below 0.50, and variance inflation factor tests confirm the absence of multicollinearity concerns because all VIF values are below 10.

Table S4. Pairwise Pearson Correlations

Variable	(1)	(2)	(3)	(4)	(5)	(6)
(1) ROA	1.000					
(2) lnTobinQ	0.147***	1.000				
(3) Ethics_Index	0.159***	-0.092***	1.000			
(4) Org_Learning	0.003	0.151***	0.176***	1.000		
(5) Tech_Complexity	-0.091***	-0.025***	0.104***	0.046***	1.000	
(6) Compliance_Cost	-0.170***	-0.091***	-0.166***	-0.078***	0.060***	1.000

Notes: *** $p < 0.01$, ** $p < 0.05$, and * $p < 0.10$.

1.6 Extended Ranking and Evaluation-Method Calibration Evidence

Table S5 retains the highest-ranked indicators from the 19-item ranking task. These rankings support the relative salience of technical robustness, privacy and data governance, algorithmic fairness, policy completeness, fundamental rights protection, human oversight, and transparency in the measurement framework.

Table S5. Highest-Ranked Indicators in the Respondent Ranking Task

Indicator	Comprehensive score
Technical robustness	13.76
Privacy and data governance	13.63
Algorithmic fairness	12.88
Policy completeness	12.60
Fundamental rights protection	12.00
Human oversight rights	11.54
Transparency	11.01
Leadership commitment	10.96
Ethics awareness maturity	10.85
Cross-departmental collaboration	10.38

Notes: Scores are from the questionnaire report's 19-indicator ranking table. The table reports the top ten indicators by comprehensive score.

Table S6 reports respondent ranking of evaluation approaches. Government compliance inspections and third-party field audits rank highest. Public annual-report text analysis ranks third. This pattern supports the study's use of public text evidence while also defining a validity boundary: annual-report scoring is auditable and scalable, but it remains less direct than regulatory inspection or field audit evidence.

Table S6. Ranking of Evaluation Approaches

Evaluation approach	Comprehensive score
Government regulatory compliance inspection results	4.23
Third-party field audit assessment	3.85
Text analysis based on public information such as annual reports	3.69
Stakeholder perception questionnaire	3.49
Media reporting and public-opinion analysis	2.98
Multiple methods used together	2.76

Notes: Scores are from the questionnaire report's evaluation-method ranking table.

The questionnaire also asked respondents to suggest thresholds for a 100-point governance evaluation system. The mean suggested pass line was 64.18 and the mean suggested excellence line was 82.12. These values are retained as interpretive calibration only. They are not used to transform the disclosure-based governance index into pass-fail classifications, because observed annual-report scores are sparse and reflect public disclosure rather than complete internal practice.

1.7 Convergent Validity Correlations

Table S7 reports Pearson correlations between the governance index and adjacent constructs over the full estimation sample. The moderate correlations with ESG ratings and digital transformation intensity support convergent validity while remaining well below levels that would indicate construct redundancy, and the horse-race regressions in the main text show that the index retains independent explanatory power.

Table S7. Convergent Validity Correlations with Adjacent Constructs

Variable	(1)	(2)	(3)	(4)	(5)	(6)
(1) Governance index	1.000					
(2) ESG rating (z)	0.382***	1.000				
(3) Digital transformation (z)	0.415***	0.128***	1.000			
(4) Patent intensity (z)	0.185***	0.092***	0.203***	1.000		
(5) Organizational learning (imputed, z)	0.218***	0.085***	0.502***	0.215***	1.000	
(6) Technical complexity (imputed, z)	0.095***	0.023***	0.332***	0.248***	0.418***	1.000

Notes: N = 27,547 firm-year observations. Organizational learning and technical complexity are the imputed regression versions, so their correlations differ from those computed on the non-imputed variables. *** $p < 0.01$.

1.8 Organizational Learning Dictionary

The organizational learning measure counts 50 dictionary terms in the MD&A section per 10,000 segmented words. The dictionary covers four term families: organizational learning and knowledge management (for example, organizational learning, knowledge management, knowledge sharing, knowledge transfer, knowledge creation, learning organization, continuous learning, experiential learning, learning by doing, learning by using, knowledge accumulation, learning curve, adaptive learning, ambidextrous learning, exploratory learning, exploitative learning, knowledge codification, knowledge storage, knowledge spillover); employee training and human capital development (employee training, human capital development, talent development, skill upgrading, capability building, mentoring systems, job-rotation learning, action learning, reflective learning, post-project review); innovation absorption and collaboration (research collaboration, technology introduction, technology absorption, technology digestion, innovation learning, industry-university-research collaboration, technology alliances, research networks, open innovation, collaborative innovation, patent citation, technology tracking, innovation culture); and continuous improvement (best practice, lessons learned, benchmarking, process improvement, quality management, six sigma, lean production, continuous improvement). The original Chinese term list is archived in the project repository and available on request; terms are reported here in English translation because the article is typeset without Chinese fonts.

1.9 Keyword-to-Indicator Mapping

The governance dictionary contains 57 keywords retained from the survey's keyword-evaluation task, three per second-level indicator. Table S8 reports the mapping in English translation; the original Chinese terms are archived in the project repository.

Table S8. Dictionary Keywords by Indicator (English Translation)

Dimension	Indicator	Keywords
Organizational governance	Ethics awareness maturity	awareness gaps; reactive response; strategic integration
	Leadership commitment	senior-executive support; budget guarantee; decision authorization
	Cross-departmental collaboration	government-enterprise collaboration; standard adoption; coordination mechanisms
	Policy completeness	guideline publication; implementation roadmap; regulatory alignment
Technical trustworthiness	Technical robustness	attack resistance; failure contingency; accuracy
	Privacy and data governance	data encryption; anonymization; access protocols
	Transparency	explainability; decision traceability; black-box avoidance
	Algorithmic fairness	bias detection; fairness testing; non-discrimination
Rights protection	Human oversight rights	human review; termination control; decision veto
	User empowerment	data erasure; appeal response; consent management
	Fundamental rights protection	privacy rights; equal treatment; rights safeguards
	Whistleblower system effectiveness	protected reporting; anonymous channels; rapid response
Social impact	Social welfare	social equity; community wellbeing; public benefit
	Environmental impact	energy consumption; carbon emissions; green computing
	Employment impact	job displacement; retraining support; workforce transition
	Malicious use prevention	deepfake prevention; misuse blocking; abuse control
Operational mechanisms	Accountability mechanisms	responsibility tracing; audit-based redress; accountability enforcement
	Risk management	full-process risk identification; tiered response; risk plans
	Ethics compliance auditing	algorithm-bias verification; privacy-compliance audit; independent review

Notes: Keywords with a mean representativeness rating below 3 in the survey's keyword-evaluation task were excluded before scoring. Keyword frequencies are counted per 10,000 segmented MD&A words.

1.10 Coding Manual Excerpt and Worked Examples

The written coding manual used for validation defines each of the 19 indicators, provides positive and negative anchor examples drawn from actual MD&A text, and specifies boundary rules for ambiguous disclosures. Two trained coders applied the manual to a stratified random validation subsample of 120 firm-year reports after a four-hour training program calibrated on twenty pilot reports; inter-rater reliability reached a mean Cohen's kappa of 0.726 across indicators (dimension-level range 0.691 to 0.758), and the eight indicator-level judgments that resisted structured consensus (0.35 percent of 2,280) were settled by the author.

Three worked examples illustrate the coding logic; the passages are paraphrased in English from actual annual-report MD&A sections, with identifying details removed.

First, a positive case. A telecommunications equipment manufacturer's 2022 report describes the release of two data-governance policy documents, a cross-departmental data ethics committee led by the chief data officer that meets quarterly to review high-risk data-processing projects, algorithmic fairness assessments completed for twelve AI application scenarios with a calibration implemented after a regional bias was detected in an intelligent customer-service system, ISO/IEC 27701 certification, an independent third-party audit of customer-data lifecycle management, full employee coverage of data-protection training, and a whistleblowing channel that handled three data-violation reports within 72 hours. This passage scores on policy completeness, leadership commitment, cross-departmental collaboration, algorithmic fairness, privacy and data governance, ethics compliance auditing, and whistleblower system effectiveness.

Second, a negative case. A construction materials firm's 2021 report states that the company values information technology, has upgraded its office systems, will explore digital transformation when conditions mature, and will continuously improve its data-security policies in line with regulatory requirements. The passage contains only aspirational language without any named policy, mechanism, metric, or governance structure, and it receives no credit on any indicator.

Third, a mixed case. A pharmaceutical firm's 2023 report documents data-verification protocols that ensure the integrity and traceability of clinical trial data, participation in an industry data-sharing alliance, and a preliminary explainability assessment process for an AI-assisted drug discovery project that has not yet been codified into formal policy. The concrete mechanisms score on technical robustness and transparency, while the preliminary explainability process is coded at reduced strength because no formal policy document exists.

1.11 Questionnaire Instrument (English Translation)

The survey instrument, the Corporate Big Data Ethics Indicator Importance Assessment Questionnaire, contains 19 items in six parts. The instrument was administered in Chinese; the item stems are reported here in English translation, and the original Chinese instrument is archived in the project repository. The archived instrument also includes the dedicated consent page shown before any survey item and the directed-response attention-check item embedded in the Likert blocks, both of which are described in the Methods section of the main text.

Part 1: Respondent information (items 1–8, single choice). (1) Gender. (2) Age bracket. (3) Highest educational attainment. (4) Job level. (5) Years of work experience in data management or digitalization. (6) Employer size (number of employees). (7) Employer's primary industry. (8) Whether the respondent is directly responsible for, or participates in, corporate data governance or privacy protection work.

Part 2: Dimension weight allocation (item 9, constant-sum). (9) Corporate big data ethics comprises five core dimensions (organizational governance, technical trustworthiness, rights protection, social impact, operational mechanisms); allocate importance weights across the five dimensions so that the total equals 100 percent.

Part 3: Second-level indicator importance (items 10–14, five-point matrix scales, 1 = very unimportant, 5 = very important). (10) Organizational governance dimension: rate the importance of ethics awareness maturity, leadership commitment, cross-departmental collaboration, and policy completeness. (11) Technical trustworthiness dimension: rate technical robustness, privacy and data governance, transparency, and algorithmic fairness. (12) Rights protection dimension: rate human oversight rights, user empowerment, fundamental rights protection, and whistleblower system effectiveness. (13) Social impact dimension: rate social welfare, environmental impact, employment impact, and malicious use prevention. (14) Operational mechanisms dimension: rate accountability mechanisms, risk management, and ethics compliance auditing.

Part 4: Text-analysis keyword evaluation (items 15–16). (15) Rate how well each candidate keyword represents the meaning of corporate big data ethics, on a five-point scale (1 = very poorly, 5 = very well); the instruction states that the keywords will be searched in corporate annual reports. (16) Open item: list three to five additional keywords that should be added to the text-analysis dictionary.

Part 5: Core indicator ranking (items 17–18, drag-and-drop ranking). (17) Rank the 19 core indicators by their importance for corporate big data ethics (1 = most important, 19 = relatively least important). (18) Rank six evaluation approaches by their effectiveness for assessing a firm's big data ethics level: government regulatory compliance inspection, third-party field audit, text analysis of public information such as annual reports, stakeholder perception surveys, media and public-opinion analysis, and combined methods (1 = most effective, 6 = relatively least effective).

Part 6: Open calibration item (item 19, slider). (19) If a 100-point corporate big data ethics evaluation system were constructed, at what score should the pass line be set, and at what score the excellence line?
